# Activation of telomerase by TA-65 enhances immunity and reduces inflammation post myocardial infarction

**DOI:** 10.1007/s11357-023-00794-6

**Published:** 2023-04-22

**Authors:** Bilal Bawamia, Luke Spray, Vincent K. Wangsaputra, Karim Bennaceur, Sharareh Vahabi, Konstantinos Stellos, Ehsan Kharatikoopaei, Emmanuel Ogundimu, Chris P. Gale, Bernard Keavney, Rebecca Maier, Helen Hancock, Gavin Richardson, David Austin, Ioakim Spyridopoulos

**Affiliations:** 1 Freeman Hospital, Newcastle Upon Tyne, UK; 2 Academic Cardiovascular Unit, The James Cook University Hospital, Middlesbrough, UK; 3 Vascular Biology and Medicine Theme, Faculty of Medical Sciences, International Centre for Life, Translational and Clinical Research InstituteNewcastle UniversityNewcastle Upon Tyne, Central Parkway, NE1 3BZ UK; 4 Faculty of Medicine, Universitas Indonesia, Central Jakarta, Indonesia; 5 Vascular Biology and Medicine Theme, Faculty of Medical Sciences, Biosciences Institute, Newcastle University, Newcastle Upon Tyne, UK; 6 Department of Cardiovascular Research, European Center for Angioscience (ECAS), Heidelberg University, Mannheim, Germany; 7 German Centre for Cardiovascular Research (DZHK), Partner Site Heidelberg/Mannheim, Mannheim, Germany; 8 Department of Cardiology, University Hospital Mannheim, Heidelberg University, Manheim, Germany; 9 Department of Mathematical Sciences, Durham University, Durham, UK; 10 Department of Cardiology, Leeds Teaching Hospitals NHS Trust, Leeds, UK; 11 Leeds Institute for Cardiovascular and Metabolic Medicine, University of Leeds, Leeds, UK; 12 Division of Cardiovascular Sciences, School of Medical Sciences, Faculty of Biology, Medicine and Health, The University of Manchester, Manchester, UK; 13 Manchester Heart Institute, Manchester University NHS Foundation Trust, Manchester Academic Health Science Centre, Manchester, UK; 14 Newcastle Clinical Trials Unit, Faculty of Medical Sciences, Newcastle University, Newcastle Upon Tyne, UK; 15 Population Health Science Institute, Newcastle University, Newcastle Upon Tyne, UK

**Keywords:** Acute myocardial infarction, Ageing, T-lymphocytes, Telomerase, Immunosenescence

## Abstract

Myocardial infarction (MI) accelerates immune ageing characterised by lymphopenia, expansion of terminally differentiated CD8^+^ T-lymphocytes (CD8^+^ T_EMRA_) and inflammation. Pre-clinical data showed that TA-65, an oral telomerase activator, reduced immune ageing and inflammation after MI. We conducted a double blinded randomised controlled pilot trial evaluating the use of TA-65 to reduce immune cell ageing in patients following MI. Ninety MI patients aged over 65 years were randomised to either TA-65 (16 mg daily) or placebo for 12 months. Peripheral blood leucocytes were analysed by flow cytometry. The pre-defined primary endpoint was the proportion of CD8^+^ T-lymphocytes which were CD8^+^ T_EMRA_ after 12 months. Secondary outcomes included high-sensitivity C-reactive protein (hsCRP) levels. Median age of participants was 71 years. Proportions of CD8^+^ T_EMRA_ did not differ after 12 months between treatment groups. There was a significant increase in mean total lymphocyte count in the TA-65 group after 12 months (estimated treatment effect: + 285 cells/μl (95% CI: 117–452 cells/ μ l, *p* < 0.004), driven by significant increases from baseline in CD3^+^, CD4^+^, and CD8^+^ T-lymphocytes, B-lymphocytes and natural killer cells. No increase in lymphocyte populations was seen in the placebo group. At 12 months, hsCRP was 62% lower in the TA-65 group compared to placebo (1.1 vs. 2.9 mg/L). Patients in the TA-65 arm experienced significantly fewer adverse events (130 vs. 185, *p* = 0.002). TA-65 did not alter CD8^+^ T_EMRA_ but increased all major lymphocyte subsets and reduced hsCRP in elderly patients with MI after 12 months.

**Supplementary Information:**

The online version contains supplementary material available at 10.1007/s11357-023-00794-6.

## Introduction

Despite modern advances in therapy, myocardial infarction (MI) still carries a substantial risk of death or recurrent cardiovascular events [1, 2], and the identification of novel mechanisms and therapeutic targets is therefore urgently needed. We have previously demonstrated that lymphopenia predicts mortality after ST-elevation MI (STEMI) [3, 4], suggesting that premature ageing of the immune system (termed “immunosenescence”) may contribute to poor outcomes.

Immunosenescence is mainly linked to thymic involution and changes in cellular immunity as a response to pathogens, such as recurrent viral infections, throughout life [5]. These include a reduction in circulating lymphocytes and naïve T lymphocytes, the loss of stimulatory T lymphocyte co-receptors, the increase of oligoclonal memory cells, and increased levels of pro-inflammatory cytokines [6]. Immune ageing has been shown to correlate with higher mortality across different age groups [7, 8]. Relative lymphopenia in over 50,000 otherwise healthy middle-aged Americans has recently been identified as a strong predictor of overall mortality as well as cardiovascular mortality [9]. We have previously shown in the Newcastle 85 + study that women exhibited higher lymphocyte counts together with a higher frequency of naïve T-cells, paralleled by lower cardiovascular mortality without differences in non-cardiovascular mortality [10, 11]. In a separate study, we have reported that myocardial infarction (MI) leads to accelerated immunosenescence and shorter leukocyte telomere length [3, 4, 12, 13].

Lymphocyte proliferation can be enhanced in vitro by activating telomerase, a reverse transcriptase that typically counteracts telomere shortening at the end of chromosomes [14]. It has become evident that telomerase is also detectable in differentiated, non-dividing, or low proliferating cells of the cardiovascular system [15]. Besides the pivotal role of its catalytic subunit telomerase reverse transcriptase (TERT) in ensuring maintenance of telomere homeostasis, evidence has accumulated demonstrating non-telomeric functions of the enzyme, owing to its localization in mitochondria. TA-65 is an Astragalus root-purified and encapsulated form of cycloastrogenol with increased bioavailability (T.A. Sciences, New York, USA). We have previously shown that TA-65 induces T-lymphocyte proliferation in a TERT-dependent way in vitro [16]. Haendeler’s group has recently shown that TA-65 is a mitochondrial rather than nuclear telomerase activator, which depends on the presence of TERT, improving the outcome of mice after experimental myocardial infarction [17].

We present the results from the Telomerase ACTivator to reverse Immunosenescence in Acute Coronary Syndrome (TACTIC) trial—the first randomized controlled trial investigating whether treatment with TA-65 for 12 months after MI reduces immune ageing and inflammation.

## Methods

### Trial design and oversight

The TACTIC trial was a single-centre, randomized, double-blind, parallel-group, placebo-controlled phase IIa pilot trial comparing TA-65 with placebo in 90 participants with coronary heart disease who were diagnosed with myocardial infarction in the 6 months prior to enrolment. A total of 90 patients were randomized to either the TA-65 group (*n* = 45) or the placebo group (*n* = 45); TA-65 and placebo were administered as 8 mg doses twice daily for 12 months. The trial was run according to the principles of Good Clinical Practice, and in accordance with all relevant UK legislation and the trial protocol. Written informed consent was obtained from all participants. TACTIC was registered at the International Standard Randomized Controlled Trial Number (ISRCTN) registry (16,613,292) and at the European Union Drug Regulating Authorities Clinical Trials Database (EudraCT), European Union Clinical Trials Register (2017–002,876-26). A favourable ethical opinion was granted on August 18^th^ 2018 by the UK Health Research Authority (18/NE/0178). The trial was funded by TA-Sciences Inc (New York, US) as an investigator-led grant to I.S.

### Trial population

Patients were eligible for the trial if they a) were aged 65 years or over with an index presentation of an acute myocardial infarction (either STEMI or NSTEMI) within the previous 6 months, b) had successfully completed revascularisation or were being managed medically following myocardial infarction, c) had evidence of obstructive coronary artery disease on invasive coronary angiography (at least one major epicardial vessel stenosis ≥ 70%), and d) were more than 24 h after presentation with the index event—patients were eligible the following day post percutaneous coronary intervention (PCI) or 3 months after CABG. Exclusion criteria included any condition associated with immunological dysfunction (acute or chronic inflammatory or neoplastic co-existing disease, known positive serology for HIV, or hepatitis); clinical instability (arrhythmias, cardiogenic shock, unconscious); severe, uncontrolled hypertension (Blood Pressure > 170/110 mmHg, or ambulatory BP of 150/95 mmHg); severe comorbidity likely to impact on outcome over the next 2 years, immunosuppressants, known malignancy, patients already taking a nutritional supplement derived from the roots of the Astragalus species, known current or previous substance addiction and insulin-dependent diabetes mellitus.

### Randomization and blinding

Randomisation was performed using a minimisation scheme to ensure patients randomised to each group were comparable at baseline. The minimisation scheme accounted for gender (male or female), type of MI (STEMI or NSTEMI) and proportion of terminally differentiated effector memory CD8^+^ T-lymphocytes (CD8^+^ T_EMRA_; high > 45% or low ≤ 45%) at baseline. Eligible patients were randomised by delegated and trained members of the research team at the site using a 24-h, central, secure, web-based randomisation system with concealed allocation. Eligible patients were randomised in a 1:1 ratio to receive TA-65 (intervention under study) or placebo (control group). Both groups also received standard NHS care for MI. Assignment to either TA-65 or placebo was blinded to the patient, treating clinicians and the clinical research team (including the pharmacy team), and staff at Newcastle Clinical Trials Unit, except for the data manager. Collection, dispensing and recalling of the drug (TA-65 or placebo) were performed by delegated members of the clinical research team. Study drugs were labelled using a unique identification code that was linked to the study randomisation system. TA-65 and its matched placebo were identical in appearance, smell and taste and presented in the same packaging to ensure blinding of the study drug.

### Outcome measures

The primary outcome measure was the proportion of CD8^+^ T-lymphocytes which were terminally differentiated effector memory cells (CD8^+^ T_EMRA_; CD3^+^CD4^−^CD8^+^CCR7^−^CD45RA^+^), measured in peripheral blood with flow cytometry at 12 months. CD8^+^ T_EMRA_ cells were chosen as a representative parameter for immune ageing, identified among others in the Newcastle 85 + study. [10] Secondary outcomes included proportions and absolute counts of other leucocytes subsets, including T-lymphocyte and monocyte subsets, serum levels of high-sensitivity C-reactive protein (hsCRP), telomerase activity, oxidative stress, microvascular endothelial function and cardiac function, assessed by both serum levels of NT-proBNP and transthoracic echocardiography, and adverse events and adverse reactions. hsCRP levels were compared between groups at 12 months. All other secondary outcome measures were analysed as the change from baseline at 12 months.

### Flow-cytometric assays

**Fig. 1:**
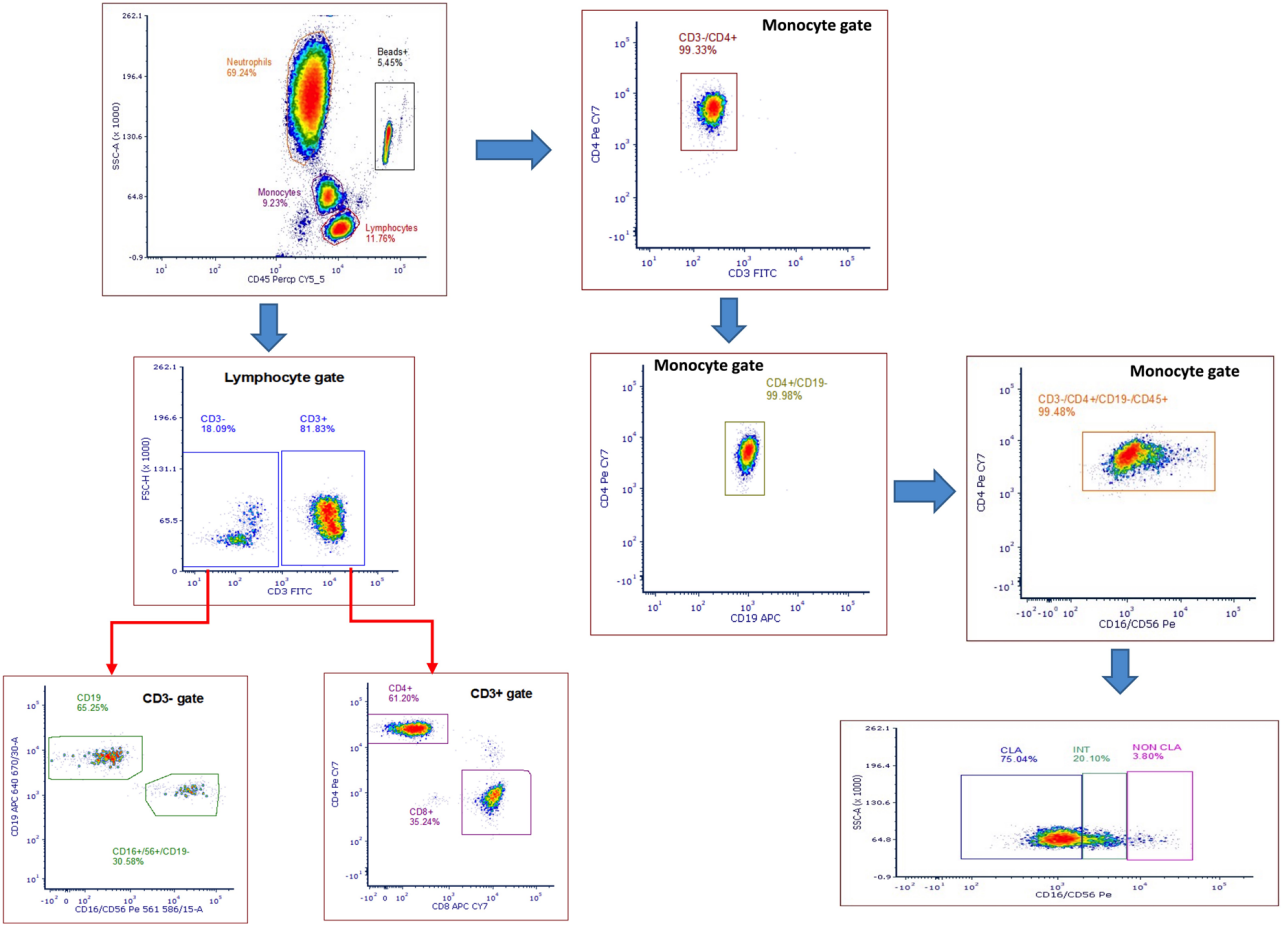
Gating of main leukocyte populations with TruCount assay. Flow cytometric analysis of a representative patient from the TACTIC trial is shown. At first, lymphocytes, monocytes, neutrophils, and beads were gated in SSC-Area against the leukocyte marker CD45. Lymphocytes were sequentially gated into CD3^+^ (T cells) and CD3^−^ cells. CD4^+^ and CD8^+^ T cells were gated from CD3 + cells, while B cells (CD19^+^) and natural killer cells (CD19^−^CD16^+^CD56^+^) were gated from CD3^−^ cells. Several sub-gates were sequentially gated from the main monocyte population to ensure that gating was performed on specific CD3^−^CD4^+^CD19^−^CD45^+^ cells. Finally, monocytes were divided into classical (CLA), intermediate (INT), or non-classical monocytes (NON CLA) based on the abundance of CD16 expression

Two different assays were used for flow cytometric analysis of leucocytes. All measurements were performed on fresh blood within 4 h of collection. There was no significant amount of non-viable cells (< 1%) with this approach. The TruCount assay (containing CD45, CD3, CD4, CD8, CD19, CD16, CD56 fluorochrome-labelled monoclonal antibodies) provided information on the absolute concentration of major leukocyte populations (Fig. 1), while the 7-colour assay (containing CD3, CD4, CD8, CD45RA, CCR7, CD28, CX3CR1 fluorochrome-labelled monoclonal antibodies) characterised a wider range of receptor expression on these cells (Supplementary Table S10 and Figure S1). By combining the absolute data of the TruCount assay with the relative data of the 7-colour FACS, we defined the size of very specific subpopulations of T-lymphocytes, including the primary outcome measure of CD8^+^ T_EMRA_. Full details of both assays are described in the Supplementary Methods. Data acquisition was performed with an Analytical Fluoresence Flow Cytometer BD FORTESSA machine using BD FACSDiva software version 8.0.1. The acquisition parameters were set to stop collecting data after 3,000 events of TruCount Beads to ensure accurate enumeration, and 10,000 CD8^+^ events for the 7-colour assay, as previously described [3].

### Telomerase activity

Nuclear telomerase activity in peripheral blood mononuclear cells (PBMCs) was measured using the Telomerase Repeated Activation Protocol (TRAP) – quantitative polymerase chain reaction (qPCR) assay. Within 24 h of collection, whole blood was centrifuged to isolate PBMCs, which were cryopreserved at − 80 C. The cells were subsequently thawed, lysed, and analysed using a validated TRAP-qPCR protocol (Supplementary Tables S11 and S12), with the human T cell leukaemia line 1301 acting as the positive control (Supplementary Figures S2 and S3) [18]. The full protocol is described in the Supplementary Methods.

### Oxidative stress

Oxidative stress was measured using the TBARS colorimetric assay. This assay is described in detail in the Supplementary Methods, but briefly involves the addition of thiobarbituric acid to plasma samples, which in the presence of lipid perioxidation products yield a pink solution, which can be quantified with colorimetry.

### Endothelial function

Endothelial function was evaluated using the EndoPAT device (Itamar Medical Ltd, Caesarea, Israel). This non-invasive operator-independent system consists of finger probes that measures peripheral arterial tone (PAT) for 5 min at rest and during reactive hyperaemia induced by 5-min forearm cuff occlusion. The reactive hyperaemia index (RHI) is the post- to pre- occlusion PAT signal ratio in the occluded arm relative to the same ratio in the control arm. RHI was measured at baseline, 6 months and 12 months (Supplementary Figure S4).

### Echocardiography

Transthoracic echocardiography (TTE) was performed at baseline and 12 months to evaluate left ventricular function including global longitudinal strain (Supplementary Methods).

### Adverse events

Adverse events (AEs), adverse reactions (ARs), serious adverse events (SAEs), and serious adverse reactions (SARs) were assessed by a medical practitioner for severity and causality. The severity of each was classified as follows: grade 1—minor event not requiring medical intervention, grade 2—an event which is symptomatic and may require medical attention, grade 3—a significant event which requires medical intervention and may require hospitalisation, grade 4—an event that requires urgent medical intervention and is potentially life-threatening, grade 5—death.

### Statistical analysis

Continuous variables were reported by number, mean ± SD, median (IQR) or minimum and maximum, whereas number and percentages summarised categorical variables. The proportion of CD8^+^ T_EMRA_ was calculated from the total number of peripheral blood CD8^+^ T cells in each blood sample, so it was treated as a continuous outcome. Therefore, a linear mixed-effect model assuming Gaussian distribution with adjustment for minimisation factors, trial group and time points (baseline and 12 months) as predictors were used for the primary analysis, using intention-to-treat principles to quantify the change in the proportion of CD8^+^ T_EMRA_ from baseline to 12 months. Continuous secondary outcomes were analysed using linear mixed-effects models to consider the follow-up time effect and intra-patient correlation. All models accounted for treatment group and stratification variables used in the randomisation scheme. The coefficients and their associated confidence intervals were reported for each predictor included in the models. All hypothesis testing was at 5% significance level. Since there was no sample size calculation, the study was not powered to detect the minimum meaningful change in the primary outcome compared to baseline. Therefore, all efficacy analyses and 95% confidence intervals are for the purpose of guidance and the focus is on the estimated effects rather than their statistical significance. All analyses were done on a PC running Microsoft Windows 10, and the statistical programming code R was used for the analysis.

## Results

### Trial progress

**Fig. 2:**
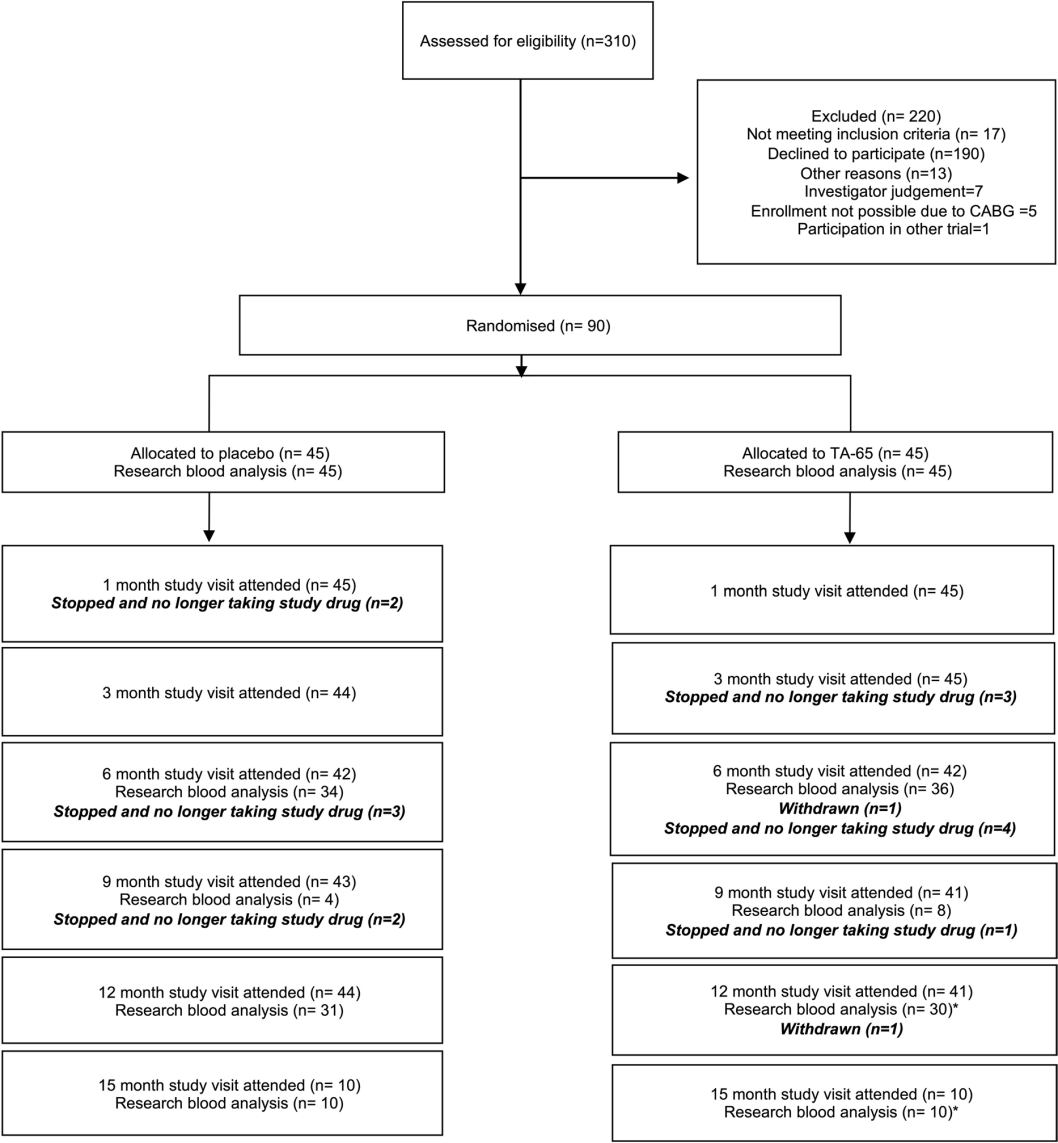
Consolidated Standards of Reporting Trials (CONSORT) diagram of patient enrolment. The diagram shows the number of patients who met the inclusion or exclusion criteria and their distribution among the two study groups. TA-65 and placebo were administered as 8-mg doses twice daily for 12 months

All patients were recruited at The James Cook University Hospital, Middlesbrough, UK, between 21^st^ January 2019 and 17^th^ March 2020. The final trial visit was performed on 18^th^ March 2021. Of 310 patients assessed for eligibility, 90 patients were randomised to placebo (*n* = 45) or TA-65 (*n* = 45; Fig. 2) and all underwent primary outcome sample analyses at baseline. No patients were lost to follow up; 15 patients (7 from placebo group and 8 from TA-65) discontinued trial medications (Supplemental Table S1) and of those, 7 attended for primary outcome sample analyses at 12 months. Due to COVID-19 related trial visit restrictions, 20 patients (10 in each group) continued trial medication for 15 months to allow measurement of final endpoints when national restrictions were lifted. 37 patients in each group of the study were adherent to treatment (pre-defined as > 80% of doses taken over the study period).

### Baseline characteristics

The median age of participants was 71 years and 16.7% were women (Table 1). All participants were of white ethnicity. There was a higher percentage of smokers or ex-smokers in the placebo group (82.2% vs 62.2%, *p* = 0.04). Both groups were otherwise well balanced with regards to baseline patient, treatment and MI characteristics. The index coronary event was NSTEMI in 52.2% and STEMI in 47.8%, and 86.7% were treated with PCI. There were 14 (31.1%) anterior infarcts in each group. Procedural characteristics are reported in Supplemental Table S2.

**Table 1:** Comparison of baseline demographics, past medical history, medications and MI characteristics between treatment groups. Continuous and categorical variables were compared using simple linear regression and logistic regression analyses respectively

	Placebo (*n* = 45)	TA-65 (*n* = 45)	*P* value
Baseline demographics			
Age, mean (SD), y	72 (4.79)	71.6 (4.83)	0.73
Male sex, n (%)	38 (84.4)	37 (82.2)	0.78
Current or ex-smoker, n (%)	37 (82.2)	28 (62.2)	0.04
Past medical history, n (%)			
Atrial fibrillation or flutter	6 (13.3)	1 (2.2)	0.08
Hypertension	21 (46.7)	24 (53.3)	0.53
Hyperlipidaemia	33 (73.3)	25 (55.6)	0.08
Type 2 diabetes mellitus	5 (11.1)	8 (17.8)	0.37
Previous MI	11 (24.4)	5 (11.1)	0.11
Previous PCI	6 (13.3)	7 (15.6)	0.76
Previous CABG	6 (13.3)	2 (4.4)	0.16
PVD	2 (4.4)	4 (8.9)	0.41
Previous TIA or CVA	5 (11.1)	2 (4.4)	0.25
CMV seropositive	34 (75.6)	31 (68.9)	0.48
Baseline medications, n (%)			
Aspirin	45 (100)	45 (100)	1
Clopidogrel	8 (18)	6 (13)	0.43
Ticagrelor	36 (80)	38 (84)	1
Prasugrel	0 (0)	1 (2)	1
Statin	44 (98)	45 (100)	0.99
ACE-inhibitor/ARB	43 (96)	43 (96)	1
Clinical measurements, mean (SD)			
Systolic BP, mmHg	119 (17)	123 (17)	0.33
Diastolic BP, mmHg	72 (11)	72 (10)	0.93
Glucose, mmol/L	6.8 (1.5)	7.1 (2.3)	0.48
Total cholesterol, mmol/L	4.5 (1.2)	4.4 (1.3)	0.61
LDL-C, mmol/L	2.5 (1.2)	2.6 (1.1)	0.63
HDL-C, mmol/L	1.3 (0.7)	1.1 (0.3)	0.16
LVEF, %	52.2 (11.4)	55.7 (10.7)	0.14
MI characteristics			
Type of MI, n (%)			
NSTEMI	23 (51.1)	24 (53.3)	-
STEMI	22 (48.9)	21 (46.7)	0.83
Anterior infarct (vs Non- Anterior), n (%)	14 (31.1)	14 (31.1)	1
MI treatment, n (%)			
PCI	41 (91.1)	37 (82.2)	-
Medical (vs PCI)	4 (8.9)	6 (13.3)	0.80
CABG (vs PCI)	0 (0)	2 (4.4)	0.46
No. of vessel disease, n (%)			
One	26 (57.8)	22 (48.9)	-
Two (vs One)	16 (35.6)	15 (33.3)	0.82
Three (vs One)	3 (6.7)	8 (17.8)	0.12

*SD* standard deviation, *MI* myocardial infarction, *PCI* percutaneous coronary intervention, *CABG* coronary artery bypass graft, *PVD* peripheral vascular disease, *TIA* transient ischemic attack, *CVA* cerebrovascular accident, *CMV* cytomegalovirus, *ACE* angiotensin-converting enzyme, *ARB* angiotensin receptor blocker, *BP* blood pressure, *LDL-C* low-density lipoprotein cholesterol, *HDL-C* high-density lipoprotein cholesterol, *LVEF* left ventricular ejection fraction, *NSTEMI* non ST segment elevation myocardial infarction, *STEMI* ST segment elevation myocardial infarction

### Effect on primary study endpoint and other leukocyte populations

**Fig. 3:**
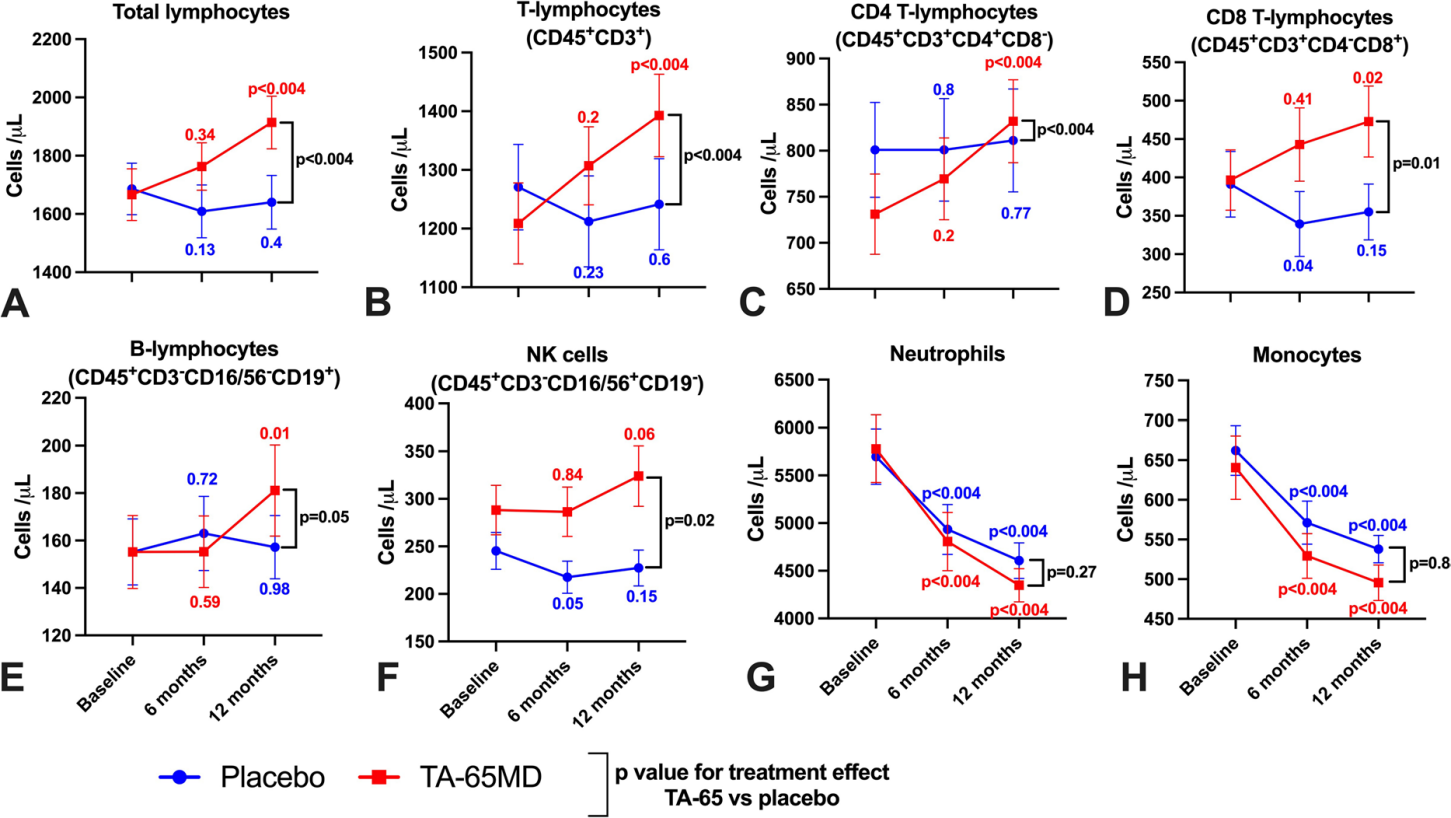
Comparison of absolute leukocyte counts (Cells/μL from Trucount assay) between TA-65 and placebo treated patients at baseline, 6 months and 12 months. (A) Total lymphocytes. (B) T-lymphocytes. (C) CD4^+^ T-lymphocytes. (D) CD8^+^ T-lymphocytes. (E) B-lymphocytes. (F) Natural Killer (NK) cells. (G) Neutrophils. (H) Monocytes.(A)–(H): Patients on TA-65 are depicted with red squares (*n* = 45 at baseline, *n* = 37 at 6 m, *n* = 40 at 12 m) and placebo-treated patients with blue circles (*n* = 45 at baseline, *n* = 36 at 6 m, *n* = 42 at 12 m). All time points are shown as mean ± standard error of mean. We performed two different statistical tests: a) 6 month and 12 month time points in each treatment arm were compared against baseline value and *p* value placed next to related data point using linear regression model (* is *p* < 0.05, ** *p* < 0.01, and *** *p* < 0.004), b) p value for treatment effect TA-65 vs. placebo after 12 months is stated next to bracket of 12 month data points using a mixed-effect linear model assuming Gaussian distribution for the error term

**Fig. 4:**
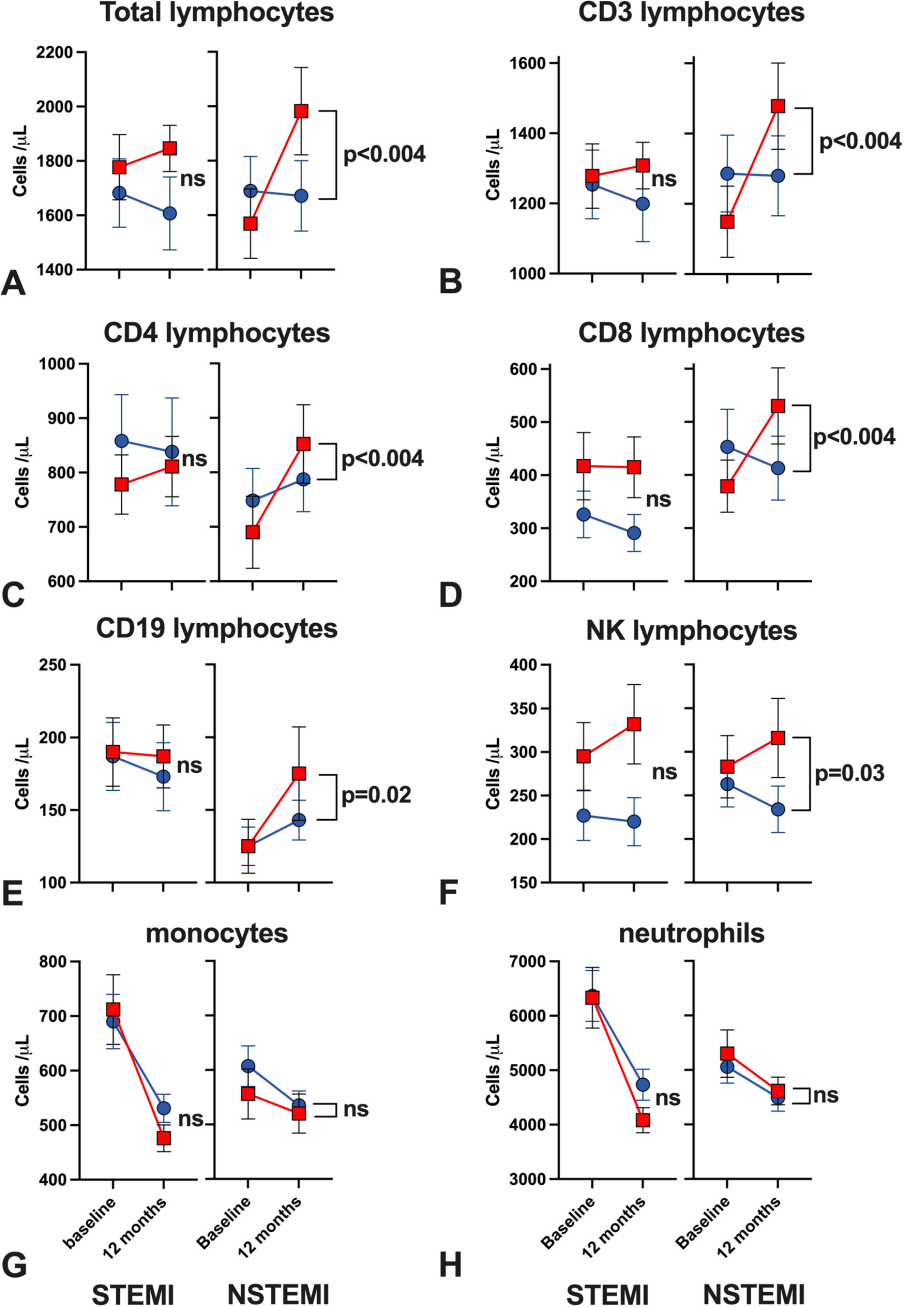
Comparison of absolute leukocyte populations (Cells/μL from Trucount assay) between TA-65 and placebo treated patients stratified to MI type at baseline and 12 months. (**A**) Total lymphocytes. (**B**) T-lymphocytes. (C) CD4^+^ T-lymphocytes. (**D**) CD8^+^ T-lymphocytes. (**E**) B-lymphocytes. (**F**) Natural Killer (NK) cells. (**G**) Neutrophils. (**H**) Monocytes. (**A**)–(**H**): Patients on TA-65 are depicted with red squares and placebo-treated patients with blue circles (left: STEMI *n* = 22 at baseline, *n* = 22 at 12 m, right: NSTEMI *n* = 21 at baseline, *n* = 20 at 12 m). All time points are shown as mean ± standard error of mean. P value for treatment effect TA-65 vs. placebo after 12 months is stated next to bracket using a mixed-effect linear model assuming Gaussian distribution for the error term. STEMI = ST-elevation myocardial infarction. NSTEMI = non-ST elevation myocardial infarction

The primary endpoint of the trial, the proportion of CD8^+^ T-lymphocytes which were CD8^+^ T_EMRA_, did not differ between the 2 groups after 12 months (Table 2). The proportion of CD8^+^ T_EMRA_ cells did not change significantly after 6 and 12 months in both groups. The proportion of CD4^+^ and CD8^+^ T cell subsets remained similar in each treatment group throughout the duration of the trial and this finding was consistent in both STEMI and NSTEMI subgroups (Supplementary Table S3 and S4). We observed a substantial, absolute increase in total lymphocyte count only in the TA-65 group (Fig. 3A). This was driven by significant increase in CD4^ +^ , CD8^+^ , B-lymphocytes and natural killer cells (Fig. 3B–F). In contrast, there were no changes in neutrophils and monocytes counts (Fig. 3G–H). The treatment effect on total lymphocyte count was estimated as + 285 cells/μl (95% CI: 117–452 cells/μl, *p* < 0.004) (Supplementary Table S5). Interestingly, this was significant in NSTEMI but not STEMI subgroups (Fig. 4). The median times (range) from MI onset to baseline blood sampling in STEMI and NSTEMI were 3 (2–111) and 19 (2–134) days respectively.

**Table 2:** Comparison of the proportion of CD4^+^ and CD8^+^ T-lymphocytes as a percentage of parent population identified as each of four subsets by flow cytometry between placebo and TA-65 is shown at baseline, 6 months and 12 months. Results are shown as the mean value (standard deviation) for each treatment arm at each time point. A linear mixed-effect model was used to quantify the change in the proportion from baseline to 12 months

		Placebo			TA-65		12-month Treatment effect	*P*-value
	Baseline Mean (SD)	6 months Mean (SD)	12 months Mean (SD)	Baseline Mean (SD)	6 months Mean (SD)	12 months Mean (SD)		
CD3^+^CD4^−^CD8^+^	24.1 (14.3)	20.9 (11.4)	20.1 (10.2)	26.9 (15.2)	26.1 (16.4)	24.4 (14.0)	-0.2 (-2.6, 2.2)	0.86
CD3^+^CD4^−^CD8^+^ Terminally differentiated effector memory *(primary endpoint)*	59.1 (19.3)	59.0 (18.5)	61.0 (19.8)	58.1 (21.7)	64.3 (18.7)	60.8 (21.0)	-0.3 (-4.1, 3.4)	0.86
CD3^+^CD4^−^CD8^+^ Naive	7.2 (6.1)	5.9 (4.3)	5.9 (4.0)	6.9 (5.1)	6.4 (4.5)	6.2 (4.4)	0.4 (-1.4, 2.2)	0.65
CD3^+^CD4^−^CD8^+^ Central memory	11.5 (7.5)	11.3 (7.0)	11.1 (7.8)	12.1 (9.8)	10.4 (7.7)	11.0 (8.4)	-0.7 (-2.5,1.1)	0.43
CD3^+^CD4^−^CD8^+^ Effector memory	22.1 (11.8)	23.8 (12.0)	22.0 (12.0)	22.9 (15.2)	18.9 (11.5)	21.9 (16.0)	0.7 (-2.5, 3.8)	0.69
CD3^+^CD4^+^CD8^−^	64.3 (14.7)	66.5 (12.7)	66.0 (12.7)	61.1 (14.8)	59.8 (16.2)	61.9 (15.1)	0.9 (-1.7,3.5)	0.49
CD3^+^CD4^+^CD8^−^ Terminally differentiated effector memory	2.6 (3.8)	2.3 (3.3)	2.6 (4.3)	3.6 (6.7)	3.6 (7.3)	3.6 (8.1)	0.2 (-0.5, 0.8)	0.60
CD3^+^CD4^+^CD8^−^ Naive	42.3 (14.3)	45.2 (15.0)	46.9 (14.2)	40.0 (15.9)	41.8 (15.0)	44.6 (15.5)	0.8 (-2.5,4.1)	0.63
CD3^+^CD4^+^CD8^−^ Central memory	42.7 (13.0)	41.5 (13.0)	39.8 (13.9)	42.4 (14.6)	42.8 (14.1)	41.3 (13.9)	-0.5 (-3.0,2.0)	0.7
CD3^+^CD4^+^CD8^−^ Effector memory	12.3 (6.9)	10.9 (6.4)	10.6 (5.9)	14.0 (13.7)	11.7 (9.5)	10.5 (9.0)	-0.5 (-2.0,1.0)	0.5

*SD* standard deviation

### Effect of TA-65 on high-sensitivity C-reactive protein

**Fig. 5:**
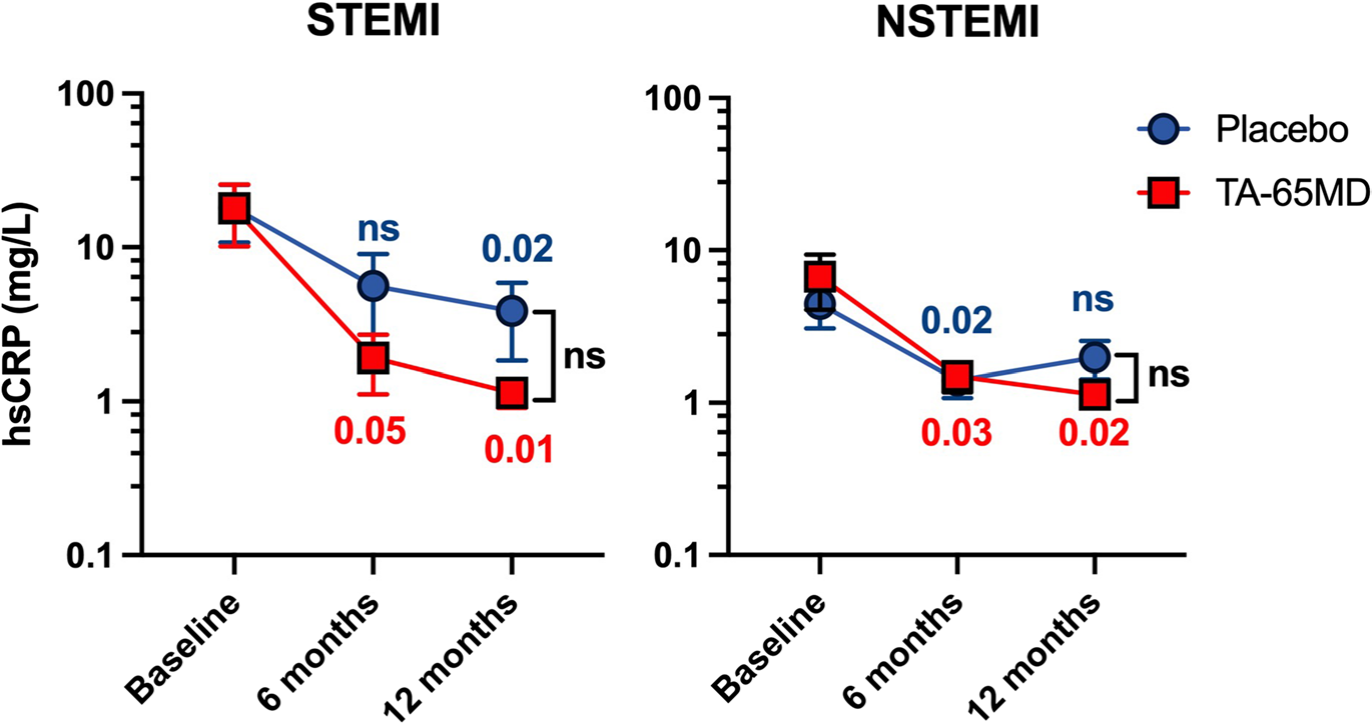
Comparison of mean hsCRP (mg/L) between TA-65 and placebo treated patients stratified to MI type at baseline, 6 months and 12 months. hsCRP is shown using a logarithmic scale. Patients on TA-65 are depicted with red squares and placebo-treated patients with blue circles. Left: STEMI patients (*n* = 20 at baseline, *n* = 17 at 6 m, *n* = 20 at 12 m), and to the right NSTEMI patients (*n* = 22 at baseline, *n* = 19 at 6 m, *n* = 20 at 12 m). All time points are shown as mean ± standard error of mean. We performed two different statistical tests: 6 month and 12 month time points in each treatment arm were compared against baseline value and *p* value placed next to related data point using linear regression model (* is *p* < 0.05, ** *p* < 0.01, and *** *p* < 0.004), b) *p* value for treatment effect TA-65 vs. placebo after 12 months is stated next to bracket of 12 month data points using a mixed-effect linear model assuming Gaussian distribution for the error term. CRP = C-reactive protein, STEMI = ST-elevation myocardial infarction. NSTEMI = non-ST elevation myocardial infarction

High-sensitivity C-reactive protein (hsCRP) levels were used as a surrogate for systemic inflammation and were similar in TA-65 and placebo groups at baseline (11.9 vs 10.9 mg/L). At 12 months, however, hsCRP levels in patients taking TA-65 were 62.1% lower than in those taking placebo (1.1 vs 2.9 mg/L). After 12 months, the treatment effect of TA-65 in change in hsCRP was estimated to be -2.9 mg/L (95% CI for treatment effect: -12.1–6.2, *p* = 0.53; Table 3). As expected, STEMI patients had higher baseline levels of hsCRP (17.9 and 18.1 mg/L for TA-65 and placebo, respectively) than NSTEMI patients (6.7 and 4.4 mg/L). In a subgroup analysis, mean hsCRP value in STEMI patients at 12 months was 71.8% lower in the TA-65 group compared to placebo (1.1 vs 3.9 mg/L); in NSTEMI patients, this was 45.0% lower (1.1 vs 2.0 mg/L) (Fig. 5). These numerical differences were not statistically significant after 12 months compared to baseline, possibly due to the low sample sizes in the subgroups. We also observed an interesting trend in the TA-65 group, where each increase in lymphocyte count of 100 cells per microliter, or 0.1 × 10^9^/mL, correlated with a 0.95 mg/L greater decrease in hsCRP between baseline and 12 months, although this correlation was not statistically significant (95% CI: − 2.28–0.42) (Supplemental Table S6).

**Table 3:** Comparison of Telomerase activity, oxidative stress, endothelial function as determined by RHI and NT pro-BNP between placebo and TA-65 is shown at baseline, 6 months and 12 months. Results are shown as the mean value (standard deviation) for each treatment arm at each time point. Secondary outcomes were analysed using linear mixed-effects models to consider the follow-up time effect and intra-patient correlation. The treatment effect represents the change in outcome due to treating with TA-65 vs placebo at 12 months

	Placebo			TA-65			12-months Treatment effect	*P*-value
	Baseline Mean (SD)	6 months Mean (SD)	12 months Mean (SD)	Baseline Mean (SD)	6 months Mean (SD)	12 months Mean (SD)		
Telomerase	N = 41	N = 36	N = 33	N = 42	N = 36	N = 34	-4.8 (-160.2,151.1)	0.95
	540.6 (378.9)	559.4 (332.1)	552.1 (269.0)	524.8 (306.1)	538.2 (347.2)	541.7 (283.3)		
Oxidative stress	N = 41	N = 35	N = 41	N = 38	N = 34	N = 39	0.08 (-0.11,0.26)	0.42
	0.69 (0.34)	0.70 (0.40)	0.60 (0.39)	0.72 (0.41)	0.77 (0.36)	0.70 (0.36)		
Endothelial function (RHI)	N = 45	N = 35	N = 37	N = 44	N = 37	N = 39	0.03 (-0.23,0.30)	0.82
	1.85 (0.51)	1.73 (0.49)	1.82 (0.51)	1.93 (0.53)	1.87 (0.35)	1.95 (0.64)		
hsCRP, mg/L	N = 42	N = 36	N = 42	N = 43	N = 36	N = 40	-2.9 (-12.1,6.2)	0.53
	10.9 (23.9)	3.4 (9.9)	2.9 (6.5)	11.9 (25.7)	1.7 (2.4)	1.1 (0.9)		
NTproBNP, pg/mL	N = 45	N = 36	N = 42	N = 45	N = 36	N = 40	-302.9 (-746.2, 141.8)	0.19
	947.4 (1084.5)	262.1 (302.7)	519.9 (1439.1)	988.1 (1408.6)	277.1 (328.8)	299.1 (348.8)		

*SD* standard deviation, *RHI* reactive hyperaemic index, *CRP* C-reactive protein, *NTproBNP* N-terminal pro B-type natriuretic peptide

### Effect on telomerase activity and oxidative stress

Nuclear telomerase activity did not change significantly between baseline and 12 months in either group. The estimated change after 12 months in telomerase activity was + 22.9 (95% CI − 91.8–136.0) in the placebo group, and + 18.5 (95% CI: − 89.4–125.9) in the TA-65 group (Table 3). Overall, there was no treatment effect at 12 months compared to baseline (− 4.8, 95% CI: − 160.2–151.1, *p* = 0.95). Treatment with TA-65 after 12 months also did not impact on oxidative stress (estimated treatment effect at 12 months compared to baseline: + 0.08, 95% CI: − 0.11–0.26, *p* = 0.42).

### Effect on endothelial function and cardiac function

There were no significant treatment effects of TA-65 on reactive hyperaemic index after 6 or 12 months compared to baseline (Table 3). NT-proBNP, a biochemical marker of ventricular stretch, reduced from 988 to 299 pg/mL after 12 months (69.7% reduction) in the TA-65 group. A smaller reduction of 45.1% was seen in the placebo group (947 to 520 pg/mL), although the treatment effect of TA-65 after 12 months was not statistically significant (Table 3). After 12 months of treatment, there were no significant treatment effects of TA-65 with respect to LV end diastolic volume, LV ejection fraction or peak systolic global longitudinal strain (Supplemental table S7).

### Adverse events and clinical outcomes

There were 315 adverse events reported, with significantly fewer in the TA-65 group compared to placebo (130 vs 185, *p* = 0.002) (Table 4). Most AEs were grade 1 (49.5%) or grade 2 (36.5%); most common were infectious, gastrointestinal or respiratory events (Supplemental Table S8). Only three AEs were possibly related to trial medication (six AEs possibly related to trial medication; Supplemental Table S9). The number of serious AEs was similar in TA-65 and placebo groups (19 vs 23); no SAEs were related to the trial medication. Fewer patients reported any grade 1–3 adverse effects in the TA-65 group (Supplemental Table 7). One patient had a grade 4 AE (respiratory failure, which resolved) and one patient died of acute myeloid leukaemia—both in the TA-65 group but were unrelated to treatment.

**Table 4:** Comparison of adverse events severity between treatment groups. Total number of adverse events compared between groups using Poisson regression. Percentages given in placebo and TA-65 columns are as proportion of all AEs of that grade. Percentage given in Total column are as percentage of all AEs. Definition of severity grade is given in the main text

Adverse Event Severity	Placebo	TA-65	Total
All Adverse Events (Grade 1–5)	185 (58.7%)	130 (41.3%)	315 (*p* = 0.002)
Grade 1	91 (58.3%)	65 (41.7%)	156 (49.5%)
Grade 2	71 (61.7%)	44 (38.3%)	115 (36.5%)
Grade 3	23 (54.8%)	19 (45.2%)	42 (13.3%)
Grade 4	0	1 (100%)	1 (0.3%)
Grade 5	0	1 (100%)	1 (0.3%)

*AE* adverse event

## Discussion

In this novel clinical trial investigating TA-65 versus placebo in patients aged 65 years and over following myocardial infarction, we established four main results: there was no significant change in senescence-like CD8^+^ T_EMRA_ after 12 months (the primary end point); there was a significant increase in lymphocytes across all major subpopulations; TA-65 was well tolerated overall with a lower number of total adverse events; there was also a trend towards less inflammation at 12 months. In brief, TA-65 is a treatment that might reduce inflammation while enhancing adaptive immunity following MI.

### Reversal of lymphopenia under TA-65

We demonstrated that TA-65 led to a reversal of relative lymphopenia. This was notable in NSTEMI but not STEMI patients. In STEMI, baseline lymphocytes are elevated between 24 and 72 h post reperfusion [3], and thereby may mask a relative increase with TA-65. The precise mechanism by which TA-65 elicits an increase in lymphocytes, paralleled by a decrease in inflammation, is unclear. TA-65 did not increase any specific lymphocyte subsets, such as T cells (increased thymus production/emigration) or B cells (enhanced emigration from bone marrow). In fact, there was no increase even in specific T cell subsets. If the treatment effect of TA-65 was due to enhanced proliferation, and therefore nuclear telomerase activation, we would expect specific subpopulations such as CD8^+^CD57^-^ to respond much stronger than their CD8^+^CD57^+^counterparts [19]. However, since naïve CD8^+^ cells (which are entirely CD57^−^) and CD8 TEMRA (or CD57^+^ subsets that are phenotypically CD28^−^CX3CR1^+^) seem to not differ in their response to TA-65, we believe that the underlying mechanism must be a different one. We have previously shown that TA-65 leads to enhanced proliferation in human and mouse T cells, but this study assessed higher doses in vitro than we would expect to generate in vivo with TA-65MD [16].

### Mitochondrial dysfunction in T cells as a driver of senescence and inflammageing

Senescence can be driven directly by mitochondrial dysfunction [20]. In fact, the mitochondrial function of various cell types, including T cells, has been shown recently to decline with increased age [21]. Callender and coworkers have shown that higher mitochondrial mass protects from senescence in CD4^+^ T cells, [22] while inhibiting mitochondrial function with rotenone generates a senescent CD8^+^ T cell phenotype, which is known to be cytotoxic and pro-inflammatory. In vivo, T cells with dysfunctional mitochondria accelerate senescence in mice, leading to a premature ageing phenotype triggered by the induction of proinflammatory cytokines (inflammageing) [23]. Senescence accumulation has been demonstrated to induce phenotypic changes in multiple cell populations associated with cardiovascular diseases, including myocardial dysfunction, atherosclerosis and hypertension [24, 25]. Recent evidence suggests that senescence is not simply a response to this disease but rather contributes to their initiation and progression, as reduction of senescence is associated with the delay, prevention, or reversal of characteristics in a variety of CVDs [24–27]. Mitochondria are necessary for the expression of the senescence-associated secretory phenotype, a pro-inflammatory cocktail by which senescent cells contribute to disease pathophysiology, directly linking mitochondrial metabolism to pro-inflammatory cellular responses [28]. As such, activation of mitochondrial TERT and improved mitochondrial function may contribute to attenuated senescence-associated secretory phenotype (SASP) production and in part explain our observations of reduced inflammation in the TA-65 treated cohort.

### Mitochondrial telomerase activation as a potential mechanism of TA-65

Although there is no direct evidence to suggest that mitochondrial Tert protects against senescence induction, global TERT activation inhibits senescence [29] and mitochondrial TERT binds to and protects mitochondrial DNA, which might help to maintain proper electron transport chain function decreasing mitochondrial reactive oxygen species (ROS), a key inducer of senescence [20, 30]. Ale-Agha et al. demonstrated the effect of TA-65 on mitochondrial, but not nuclear, telomerase and showed that mitochondrial TERT reduced infarct size after experimental MI in mice [17]. Mitochondria are necessary for the expression of SASP, a pro-inflammatory cocktail by which senescent cells contribute to disease pathophysiology, directly linking mitochondrial metabolism to pro-inflammatory cellular responses [28]. As such, activation of mitochondrial TERT and improved mitochondrial function may contribute to attenuated SASP production, and in part explain our observations of reduced inflammation in the TA-65 treated cohort [31]. Further high-resolution immunophenotyping, such as through spectral or mass cytometry, needs to be carried out to unravel a potential mechanistic link between the increase in lymphocyte populations and reduction in inflammation [32]. Hence, we speculate that TA-65 is a mitochondrial telomerase activator and enhances mitochondrial function to reduce inflammation [20]. In fact, our data did not show differences in nuclear telomerase activity between patient arms. Mitochondrial TERT appears to influence mitochondrial biogenesis and metabolism, which is likely to affect survival of cells as well as a reduction in SASP characterised by secretion of pro-inflammatory cytokines. Acute and chronic inflammation lead to retainment of lymphocytes in either lymphatic tissues, adherence to endothelium in venous circulation, or transmigration to inflamed tissues [33]. Reduced inflammation may therefore independently contribute to a higher number of lymphocytes in the circulation.

### Inflammation as a residual risk post ACS

Secondary prevention in patients who have undergone revascularisation after myocardial infarction aims to reduce their residual risk, predominantly by targeting hypercholesterolemia (with cholesterol-lowering agents) and platelet aggregation (with antiplatelet therapy). Recently, inflammation, as quantified by hsCRP, has been added to these determinants of residual risk. The CANTOS trial demonstrated that reducing inflammation in patients with coronary artery disease and elevated hsCRP (> 2 mg/l) reduced the rate of major adverse cardiovascular events [34]. In a more detailed subanalysis of the CANTOS trial, the authors found that the relative reduction in cardiovascular event rate was greatest in patients with the greatest on-treatment reduction in hsCRP [35]. Interestingly, a reduction in hsCRP to below 1.8 mg/L reclassified patients into a lower risk group. In the TACTIC trial, we observed that the average hsCRP in our placebo group at 12 months was 2.9 mg/L, which could be considered as high risk, but was 62% lower in those treated with TA-65, at 1.1 mg/L, which would be low risk. The treatment effect was not statistically significant as the trial was not powered to detect a difference in hsCRP and there was a large variability of hsCRP in the acute phase of MI. The reduction in hsCRP with TA-65 was even more pronounced in STEMI patients, at 72%. Reducing inflammation after myocardial infarction is expected to yield benefit in two major ways. Firstly, myocardial infarction has been shown to accelerate the progression of coronary atherosclerosis even in the non-culprit artery, possibly through focal arterial inflammation, and reducing inflammation is expected to delay this process [36, 37]. Secondly, adverse LV remodelling is propagated by excessive inflammation, and early studies with the IL-1 antagonist anakinra following MI suggest a reduction in incidence of heart failure [38]. In the TACTIC trial, NT-proBNP was lower in the TA-65 group after 12 months (299 vs 520 pg/mL), but the treatment effect was not statistically significant.

The major limitation of canakinumab in the CANTOS trial was the effect of IL-1β blockade on immunity. Patients in the treatment group had more infections, and a higher risk of dying from sepsis. However, our data shows that patients treated with TA-65 have no significant change in myeloid cells (monocytes and neutrophils) compared to placebo, suggesting they do not have impaired innate immunity. Indeed, their adaptive immunity appears to be enhanced, as indicated by an increase in the number of all lymphocyte subsets. An increase in lymphocyte count from 1600 to 1900 cells/µL (similar to what we have observed with TA-65 treatment in this study) correlates with a 50% reduction in the hazard ratio for both all-cause mortality and cardiovascular mortality in the large JAMA cohort study of 50,000 healthy Americans [9].

In summary, the telomerase activator TA-65 appears to enhance adaptive immune cell numbers in the peripheral blood without negatively affecting innate immunity. We observed a trend towards reduced inflammation in patients following MI, and we hypothesise based on work by other groups that improved mitochondrial function in T lymphocytes leads to a reduction in cytokine generation and hence reduced inflammation. Clinically, there was no evidence for an increase in adverse events with TA-65 – in fact there were significantly fewer adverse events in the TA-65 group.

## Limitations

Our trial did not have an age matched healthy control. It is not clear whether TA-65 preserves lymphocyte count for the age group or accelerates its return to normal after MI. While a reduction in hsCRP is demonstrated, measurement of systemic cytokine levels is needed to elucidate the impact of TA-65 on SASP. We used the Becton Dickinson 6-colour Trucount assay which has premixed antibodies that have been tested vigorously in clinical settings. It only comes with CD16, and not CD14. This limits our interpretation of monocyte subtypes as CD16 alone is not a fully reliable marker to distinguish intermediate, non-classical and classical monocytes. The mechanism of TA-65 remains unexplained. We plan to explore potential mechanisms in future studies by measuring telomere DNA length and mitochondrial functional assays in stored PMBC samples. Finally, the effects of TA-65 on lymphocyte count elevation and hsCRP reduction warrant an adequately powered study.

## Conclusion

Our pilot trial suggests that activation of telomerase could be a promising new drug target for patients following MI. Given the significant findings in lymphocyte increase, the trend in hsCRP reduction, as well as the extremely favourable safety profile, we propose as a next step a larger phase II clinical trial with multiple centres. We would set out to confirm whether one-year treatment with TA-65 following myocardial infarction can reduce hsCRP, shifting patients from a high to a low risk inflammatory profile.

### Supplementary Information

Below is the link to the electronic supplementary material.

Supplementary file1 (DOCX 11827 KB)

## Data Availability

Data are available upon request and following approval by the trial steering committee.

## Abbreviations:

CABG: Coronary artery bypass graft
MI: Myocardial infarction
PAT: Peripheral arterial tone
PCI: Percutaneous coronary intervention
RHI: Reactive hyperaemia index
STEMI: ST segment elevation myocardial infarction
TERT: Telomerase reverse transcriptase
