# Supplementary material for: Activation of telomerase by TA-65 enhances immunity and reduces inflammation post myocardial infarction
Source: GeroScience. 2023 Apr 22;45(4):2689–705. doi: 10.1007/s11357-023-00794-6 (PMC10122201; doi:10.1007/s11357-023-00794-6)
Supplement: Supplementary file 1 — Supplementary file1 (DOCX 11827 KB) [file 11357_2023_794_MOESM1_ESM.docx]

**Supplement Results, Bawamia et al.**

**Supplemental Table S1: Reasons for stopping IMP before schedule**. IMP= Investigational Medicinal Product; IDMEC = independent data monitoring and events committee.

| Reasons for stopping IMP | Notes |
| --- | --- |
| *Patient decision*  Adverse Event – 5  Personal choice – 5  Other – 1 | AE included joint pain, chest infection, pulmonary embolism, nausea, proctitis |
| *Principal investigator decision* Cancer diagnosis – 3 |  |
| *IDMEC*  Inclusion criteria not met – 1 |  |

**Supplemental Table S2: Procedure details for percutaneous coronary intervention performed in TA-65 and placebo arms.** Data given with medial and IQR (interquartile range) or mean and SD (standard deviation).

|  | **Placebo**  **(N=41)** | **TA-65**  **(N=37)** |
| --- | --- | --- |
| No. of vessels attempted, median (IQR) | 1 (0) | 1 (0) |
| No. of lesions attempted, median (IQR) | 1 (0) | 1 (0) |
| No. of drug eluting stents, median (IQR) | 1 (1) | 2 (1) |
| Maximum target vessel diameter, mean (SD), mm | 3.6 (0.7) | 3.7 (0.6) |
| Total stent length, mean (SD), mm | 36.7 (22.3) | 43.3 (23.7) |

**Table S3. Proportion of CD4^+^ and CD8^+^ T-lymphocytes as a percentage of parent population identified as each of four subsets by flow cytometry in ST-segment elevation myocardial infarction subgroup.** Results shown as the mean and SD (standard deviation) for each treatment arm at baseline, 6 months and 12 months. A linear mixed-effect model was used to quantify the change in the proportion from baseline to 12 months.

|  |  | **Placebo** | |  | **TA-65** | | **12-month Treatment effect** | **P-value** |
| --- | --- | --- | --- | --- | --- | --- | --- | --- |
|  | **Baseline**  **Mean (SD)** | **6 months**  **Mean (SD)** | **12 months**  **Mean (SD)** | **Baseline**  **Mean (SD)** | **6 months**  **Mean (SD)** | **12 months**  **Mean (SD)** |  |  |
| **CD3^+^CD4^-^CD8^+^** | 20.6 (13.0) | 18.9 (10.5) | 17.7 (9.0) | 25.9 (14.8) | 23.5 (15.3) | 23.2 (13.3) | 0.6 (-3.1,4.3) | 0.75 |
| CD3^+^CD4^-^CD8^+^ Terminally differentiated effector memory *(primary endpoint)* | 58.3 (17.7) | 61.2 (18.8) | 61.6 (18.8) | 54.2 (21.7) | 61.8 (18.7) | 59.9 (19.9) | 1.4 (-3.5, 6.3) | 0.58 |
| CD3^+^CD4^-^CD8^+^ Naive | 7.1 (4.6) | 5.4 (3.0) | 5.8 (3.7) | 8.2 (3.6) | 8.6 (3.8) | 7.9 (4.9) | 0.8 (-1.2, 2.9) | 0.42 |
| CD3^+^CD4^-^CD8^+^ Central memory | 12.1 (6.7) | 11.0 (6.6) | 10.8 (6.1) | 14.1 (9.5) | 12.3 (7.6) | 12.6 (8.9) | -0.2 (-2.5,2.0) | 0.84 |
| CD3^+^CD4^-^CD8^+^ Effector memory | 22.6 (11.1) | 22.3 (11.6) | 21.8 (12.2) | 23.4 (15.4) | 17.2 (10.8) | 19.6 (14.1) | -2.1 (-5.9,1.6) | 0.28 |
| **CD3^+^CD4^+^CD8^-^** | 67.3 (14.0) | 69.2 (12.3) | 68.6 (12.7) | 62.5 (15.6) | 63.6 (16.5) | 63.6 (15.3) | -0.2 (-3.9,3.4) | 0.91 |
| CD3^+^CD4^+^CD8^-^ Terminally differentiated effector memory | 1.4 (2.4) | 1.0 (1.2) | 1.8 (4.0) | 3.5 (4.1) | 3.7 (3.8) | 3.5 (4.4) | -0.1 (-0.9, 0.7) | 0.87 |
| CD3^+^CD4^+^CD8^-^ Naive | 43.6 (15.3) | 47.0 (14.7) | 46.0 (16.0) | 40.3 (13.9) | 43.5 (12.6) | 45.3 (14.7) | 3.1 (-2.0, 8.2) | 0.25 |
| CD3^+^CD4^+^CD8^-^ Central memory | 45.3 (13.9) | 43.1 (13.3) | 42.8 (14.9) | 44.3 (13.4) | 42.6 (12.5) | 41.8 (13.4) | -1.3 (-5.4,2.8) | 0.53 |
| CD3^+^CD4^+^CD8^-^ Effector memory | 9.6 (4.9) | 8.9 (5.1) | 9.4 (5.8) | 11.8 (9.6) | 10.1 (9.6) | 9.4 (7.8) | -1.6 (-3.7, 0.4) | 0.13 |

**Table S4. Proportion of CD4^+^ and CD8^+^ T-lymphocytes as a percentage of parent population identified as each of four subsets by flow cytometry in NonST-segment elevation myocardial infarction subgroup subgroup.** Results shown as the mean and SD (standard deviation) for each treatment arm at baseline, 6 months and 12 months. A linear mixed-effect model was used to quantify the change in the proportion from baseline to 12 months.

|  |  | **Placebo** | |  | **TA-65** | | **12-month Treatment effect** | **P-value** |
| --- | --- | --- | --- | --- | --- | --- | --- | --- |
|  | **Baseline**  **Mean (SD)** | **6 months**  **Mean (SD)** | **12 months**  **Mean (SD)** | **Baseline**  **Mean (SD)** | **6 months**  **Mean (SD)** | **12 months**  **Mean (SD)** |  |  |
| **CD3^+^CD4^-^CD8^+^** | 27.5 (15.0) | 22.7 (12.1) | 22.3 (11.1) | 27.9 (15.8) | 28.6 (17.4) | 25.7 (15.0) | -1.1 (-4.2, 2.1) | 0.50 |
| CD3^+^CD4^-^CD8^+^ Terminally differentiated effector memory *(primary endpoint)* | 59.9 (21.1) | 56.9 (18.5) | 60.4 (21.1) | 61.5 (21.5) | 66.5 (18.8) | 61.8 (22.6) | -2.1 (-7.6, 3.5) | 0.47 |
| CD3^+^CD4^-^CD8^+^ Naive | 7.4 (7.4) | 6.3 (5.2) | 6.0 (4.4) | 5.7 (5.9) | 4.4 (4.2) | 4.4 (3.2) | 0.0 (-2.9, 3.0) | 0.98 |
| CD3^+^CD4^-^CD8^+^ Central memory | 11.0 (8.3) | 11.6 (7.5) | 11.4 (9.3) | 10.4 (10.0) | 8.6 (7.5) | 9.5 (7.9) | -1.2 (-4.0, 1.5) | 0.39 |
| CD3^+^CD4^-^CD8^+^ Effector memory | 21.7 (12.7) | 25.1 (12.5) | 22.1 (12.2) | 22.4 (15.3) | 20.4 (12.1) | 24.3 (17.7) | 3.4 (-1.5, 8.2) | 0.18 |
| **CD3^+^CD4^+^CD8^-^** | 61.4 (15.1) | 64.2 (13.0) | 63.5 (12.4) | 59.8 (14.3) | 56.2 (15.6) | 60.2 (15.1) | 2.0 (-1.7, 5.7) | 0.29 |
| CD3^+^CD4^+^CD8^-^ Terminally differentiated effector memory | 3.8 (4.6) | 3.5 (4.1) | 3.3 (4.6) | 3.6 (8.5) | 3.5 (9.7) | 3.8 (10.7) | 0.4 (-0.6, 1.4) | 0.44 |
| CD3^+^CD4^+^CD8^-^ Naive | 41.1 (13.4) | 43.6 (15.5) | 47.9 (12.7) | 39.7 (17.8) | 40.2 (17.1) | 43.8 (16.6) | -1.2 (-5.4, 2.9) | 0.56 |
| CD3^+^CD4^+^CD8^-^ Central memory | 45.3 (13.9) | 43.1 (13.3) | 42.8 (14.9) | 44.3 (13.4) | 42.6 (12.5) | 41.8 (13.4) | -1.3 (-5.4, 2.8) | 0.53 |
| CD3^+^CD4^+^CD8^-^ Effector memory | 14.8 (7.7) | 12.8 (6.9) | 11.7 (5.8) | 15.9 (16.5) | 13.2 (9.4) | 11.7 (10.2) | 0.6 (-1.6,2.8) | 0.59 |

**Supplemental Table S5 – Raw data showing the absolute counts of major leucocyte populations as measured by TruCount flow cytometry.** Results shown as the mean and standard deviation (SD) for each treatment arm at baseline, 6 months and 12 months. A linear mixed-effect model was used to quantify the change in absolute lymphocyte counts from baseline to 12 months.

| Cell type, cells/µL (SD) | **Placebo** | | | **TA-65** | | | **12-month Treatment effect** | **P value** |
| --- | --- | --- | --- | --- | --- | --- | --- | --- |
|  | **Baseline**  **N=45** | **6 months**  **N=36** | **12 months**  **N=42** | **Baseline**  **N=45** | **6 months**  **N=37** | **12 months**  **N=40** |  |  |
| **Lymphocytes** | 1686.0 (591.2) | 1609.1 (543.8) | 1640.6 (596.1) | 1666.3 (594.1) | 1762.9 (495.4) | 1914 (571.8) | 284.6 (117.1,452.3) | <0.004 |
| T-lymphocytes: CD3^+^ | 1270.7 (488.3) | 1212.3 (465.6) | 1241.5 (504.1) | 1208.8 (462.0) | 1307.0 (405.1) | 1392.8 (444.0) | 189.8 (66.3,313.9) | <0.004 |
| CD3^+^CD4^+^CD8^-^ | 801.9 (345.4) | 800.9 (333.7) | 811.2 (361.9) | 731.2 (291.9) | 769.5 (269.9) | 831.9 (284.6) | 94.0 (20.7, 167.3) | 0.01 |
| CD3^+^CD4^+^CD8^-^CD28^-^ | 53.5 (101.8) | 41.8 (55.1) | 48.5 (76.4) | 37.2 (59.3) | 46.0 (67.4) | 50.6 (84.5) | 15.4 (1.0,29.9) | 0.04 |
| CD3^+^CD4^-^CD8^+^ | 391.1 (286.7) | 339.4 (253.8) | 355.0 (236.4) | 396.7 (263.0) | 442.9 (290.5) | 472.9 (292.1) | 77.6 (23.6,132.1) | 0.01 |
| CD3^+^CD4^-^CD8^+^CD28^-^ | 64.7 (19.6) | 64.9 (19.1) | 65.8 (19.7) | 58.2 (22.8) | 64.9 (18.5) | 64.0 (21.1) | 2.2 (-2.1,6.6) | 0.32 |
| NK cells: CD3^-^CD16^+^CD56^+^ | 245.3 (129.5) | 217.6 (101.7) | 227.3 (122.8) | 288.2 (174.5) | 286.4 (157.6) | 324.0 (201.3) | 67.4 (12.2,122.0) | 0.02 |
| B-lymphocytes: CD3^-^CD19^+^ | 155.2 (93.3) | 163 (93.9) | 157.2 (86.5) | 155.2 (103.1) | 155.3 (91.6) | 181.1 (121.4) | 21.7 (0.1,43.3) | 0.05 |
| **Monocytes:** CD3^-^CD4^+^CD19^-^CD45^+^ | 662.1 (209.4) | 571.2 (162.5) | 537.8 (111.6) | 640.6 (267.5) | 529.3 (171.1) | 495.5 (141.5) | -12.8 (-110.8,84.7) | 0.80 |
| Classical: CD3^-^CD4^+^CD19^-^CD45^+^CD16^low^ | 532.0 (177.9) | 460.9 (149.5) | 442.0 (99.7) | 523.3 (230.5) | 462.7 (221.1) | 407.3 (111.6) | -22.1 (-109.0,64.0) | 0.62 |
| Non-classical: CD3^-^CD4^+^CD19^-^CD45^+^CD16^high^ | 25.5 (23.5) | 17.7 (27.6) | 14.4 (21.6) | 22.2 (27.6) | 19.7 (24.3) | 14.6 (20.7) | 2.6 (-6.9,12.2) | 0.60 |
| Intermediate: CD3^-^CD4^+^CD19^-^CD45^+^CD16^int^ | 89.9 (48.7) | 83.8 (47.8) | 76.0 (44.4) | 82.8 (48.8) | 87.0 (59.9) | 75.8 (47.6) | 5.9 (-12.7,24.4) | 0.54 |
| **Neutrophils** | 5696.5 (1941.4) | 4933.8 (1566.6) | 4606.8 (1209.2) | 5778.9 (2365.1) | 4806.6 (1856.3) | 4348.7 (1101.4) | -445.3 (-1228.1,344.0) | 0.27 |

**Supplemental Table S6: Effect of lymphocyte count on change in High-sensitivity CRP (hsCRP) between baseline and 12 months.** In the placebo group, increased lymphocyte count was not associated with reduction in hsCRP. In the TA-65 group, a one unit (cell/microliter) increase in total lymphocyte count was associated with a 0.0095mg/L reduction in hsCRP, although this was not statistically significant.

| **High-sensitivity CRP** | | | | | | | | |
| --- | --- | --- | --- | --- | --- | --- | --- | --- |
| **Time Points** | **Placebo** | | | **TA-65** | | | **Treatment effect** | |
|  | n | Mean(SD) | Change from Baseline for one unit increase in Lymphocytes (95% CI) | n | Mean(SD) | Change from Baseline for one unit increase in Lymphocytes (95% CI) | n | Change from Baseline for one unit increase in Lymphocytes Diff(95% CI) |
| Baseline | 42 | 10.93 (23.92) | - | 43 | 11.92 (25.69) | - | 85 | - |
| 12 month | 42 | 2.87 (6.46) | 0.0001 (-0.0118 – 0.012) | 40 | 1.13 (0.94) | -0.0095 (-0.023 – 0.004) | 82 | -0.009 (-0.027 – 0.009) |

**Supplemental Table S7: Comparison of adverse event severity per patient between treatment groups.** The number of patients in each treatment group reporting at least 1 adverse event of each grade.

| **Adverse Event Severity** | **Placebo (n=45)** | **TA-65 (n=45)** |
| --- | --- | --- |
| Grade 1 | 36 (80%) | 31(68.9%) |
| Grade 2 | 27 (60%) | 24 (53.3%) |
| Grade 3 | 10 (22.2%) | 7 (15.6%) |
| Grade 4 | 0 (0%) | 1 (2.2%) |
| Grade 5 | 0 (0%) | 1 (2.2%) |

**Supplemental Table S7: Summary of echocardiographic parameters in placebo and TA-65 arms at baseline and 12 months.** Number of patients given in column headings accurate for echocardiographic measurements. LV= Left ventricle.

|  | **Placebo** | | **TA-65** | | **Treatment effect** |
| --- | --- | --- | --- | --- | --- |
| Mean (SD) | **Baseline**  **N=44** | **12 months**  **N=39** | **Baseline**  **N=44** | **12 months**  **N=36** |  |
| LV end systolic dimension (M-mode), cm | 3.4 (0.7) | 3.5 (0.8) | 3.1 (0.8) | 3.3 (0.7) | 0.1 (-0.2, 0.5) |
| LV end diastolic dimension (M-mode), cm | 4.7 (0.5) | 4.7 (0.6) | 4.6 (0.5) | 4.6 (0.7) | 0.0 (-0.3, 0.3) |
| LV end systolic volume, mL | 39.2 (15.7) | 39.9 (18.8) | 38.4 (18.0) | 38.2 (15.6) | 3.7 (-2.8,9.8) |
| LV end diastolic volume, mL | 86.2 (21.8) | 89.0 (24.2) | 86.4 (24.2) | 83.8 (28.8) | -1.4 (-13.1,9.6) |
| LV ejection fraction, % | 52.2 (11.4) | 54.2 (11.2) | 55.7 (10.7) | 56.0 (7.6) | -2.4 (-6.3,1.6) |
| Peak systolic global longitudinal strain, % | 76 | -16.4 (4.0) | -16.5 (7.4) | -18.4 (4.2) | -0.1 (-2.3, 2.0) |

**Supplemental Table S8: Total number of adverse events classified according to organ system.**

| Organ system | Number |
| --- | --- |
| Cardiac | 35 |
| Respiratory | 41 |
| Vascular | 11 |
| Gastrointestinal | 38 |
| Infection | 46 |
| Nervous | 19 |
| Musculoskeletal | 38 |
| Skin | 20 |
| Psychiatric | 15 |
| Blood and lymphatics | 13 |
| Renal | 6 |
| Other | 33 |
| **Total** | **315** |

**Supplemental Table S9: Summary of adverse event causality.** Of 9 adverse events possibly related to Investigational Medicinal Product, 8 were grade 1 severity and 1 was grade 3 severity (hospitalisation due to severe anxiety).

| **Adverse Event Causality** | **Placebo (n=185)** | **TA-65 (n=130)** | **Total (n=315)** |
| --- | --- | --- | --- |
| Possibly related | 6 | 3 | 9 |
| Unlikely to be related | 17 | 6 | 23 |
| Unrelated | 162 | 121 | 283 |

**Supplemental Methods**

**Flow cytometry analysis**

Two different assays were used in this study for flow cytometric analysis of leucocytes.

TruCount provided information on the absolute concentration of major leucocyte populations, while the 7-colour assay characterised a wider range receptor expression on these cells. Immediately after arrival, EDTA samples were placed on a Stuart Roller Mixer SRT6 until analysis. All incubation steps were carried out in the dark at room temperature.

***TruCount assay***

TruCount tubes contain a known number of fluorescent beads, allowing for precise calculation of the absolute count of a cell of interest with flow cytometry (**Equation 1**). Our antibody mix allowed calculation of absolute cells counts of CD45^+^ (leucocytes), CD19^+^ (B-lymphocytes), CD16/56^+^ (Natural Killer cells), CD3^+^ (T-lymphocytes), and CD4^+^ and CD8^+^ cells.

Absolute count of CD3^+^cells/μL

Number of CD3^+^cell events recorded

Number of bead events recorded

Number of beads per test tube

Sample volume (μL)

X

=

**Equation 1** – Calculation of absolute count of CD3^+^ cells using fluorescent beads in the TruCount assay

From each EDTA blood sample, 100μL was aliquoted and added to the bottom of a BD TruCount tube (340334, BD Biosciences) using a calibrated air displacement P200 pipette. 20μL of BD Multitest 6-Color TBNK reagent (644611, BD Biosciences) was deposited near the blood, on the side of the tube, ensuring no contact was made with the sample. The BD Multitest 6-Color TBNK consisted of the following antibodies, in buffer with 0.1% sodium azide:

- CD3 Fluorescein isothiocyanate (FITC)
- CD16 Phycoerythrin (PE)
- CD56-PE
- CD45-Per-Cy5.5
- CD4-PE-Cy7
- CD19 Allophycocyanin (APC)
- CD8-APC-Cy7

After vortexing the test sample using a Vortex Genie 2 (Scientific industries), the mixture was incubated for 30 minutes. Erythrocytes were lysed by adding 2mL of fresh lysis buffer 1x concentration, prepared using BD Pharm Lyse Lysing Buffer (10x Conc. 5075567, BD Biosciences) and Gibco distilled water (15230-147, Life technologies). After vortexing, and another 30 minutes of incubation, the samples were run using a BD LSRFortessa cell analyser (BD Biosciences, serial number: H647177E1001) until 3000 bead events were recorded, and data was extracted with BD FACSDiva Software.

***7-colour Fluorescence cytometry assay***

Multicolour flow cytometry was used to characterise the expression of a range of cell-surface receptors on T-lymphocytes. Three receptors analysed in this assay were also analysed with TruCount (CD3, CD4 and CD8) and some were not (CD45RA, CCR7, CX_3_CR1 and CD28). By combining the absolute data of TruCount with the relative data of FACS, we were able to define the size of very specific subpopulations of T-lymphocytes.

For each EDTA sample, 100µL of fresh blood was aliquoted into Falcon® Round-Bottom Polystyrene FACS tube (352054, BD Biosciences). A cocktail of antibodies was added to the sample, as described in **Table S10**. The tube was vortexed and incubated for 30 minutes. 2ml of lysis buffer 1x concentrate (as prepared for the TruCount assay) was added and the tube was vortexed, then incubated for 30 minutes. The sample was then washed twice with PBS. Following each wash, the tube was centrifuged at 400G for 5 minutes and the supernatant was removed. Sample analysis and data acquisition was performed with the same machine and software as for the TruCount assay. The sample was run to 10,000 CD8^+^ events. An example gating strategy is shown in **Figure S1**.

**Figure S1 - Gating strategy of 7-colour FACS.** Data shown for a representative patient, TA-112. Lymphocytes were gated by FSC and SSC. CD3^+^ lymphocytes were then subsequently gated into CD4^+^ and CD8^+^ T cells. Four subpopulations of CD4+ and CD8+ T cells (T_N_, T_CM_, T_EM_ and T_EMRA_) were characterised by expression of CCR7 and CD45RA. CD28/CX3CR1 density plots were set for CD4^+^ and CD8^+^ T cells along with their subsets.


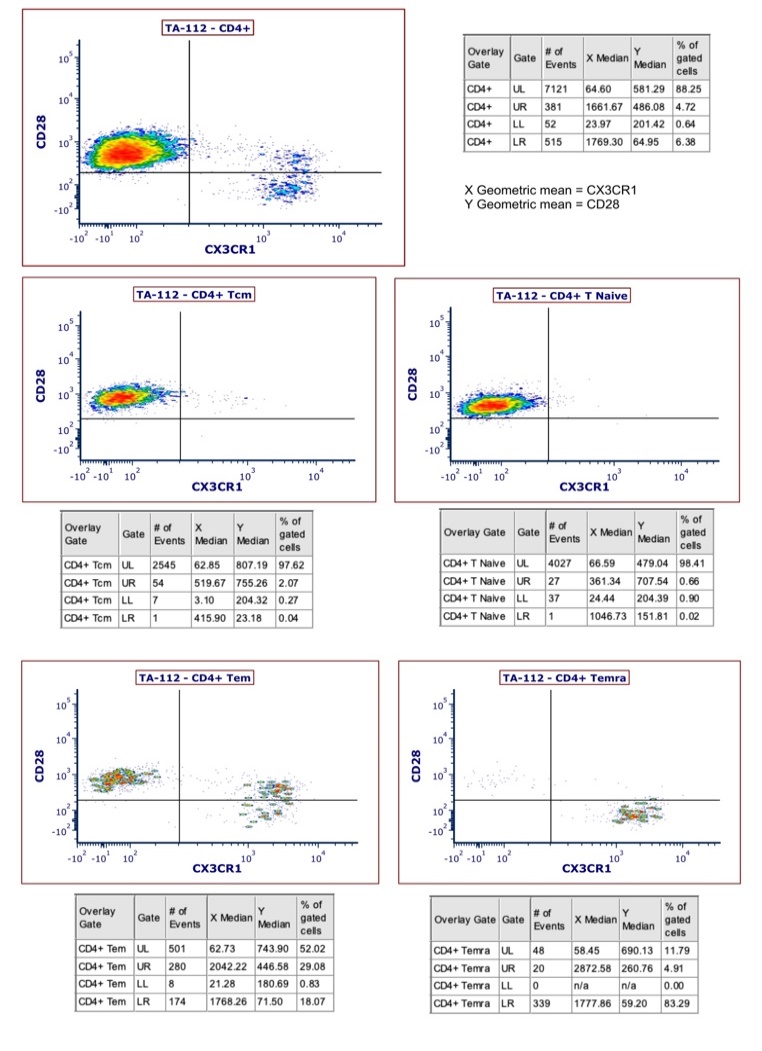

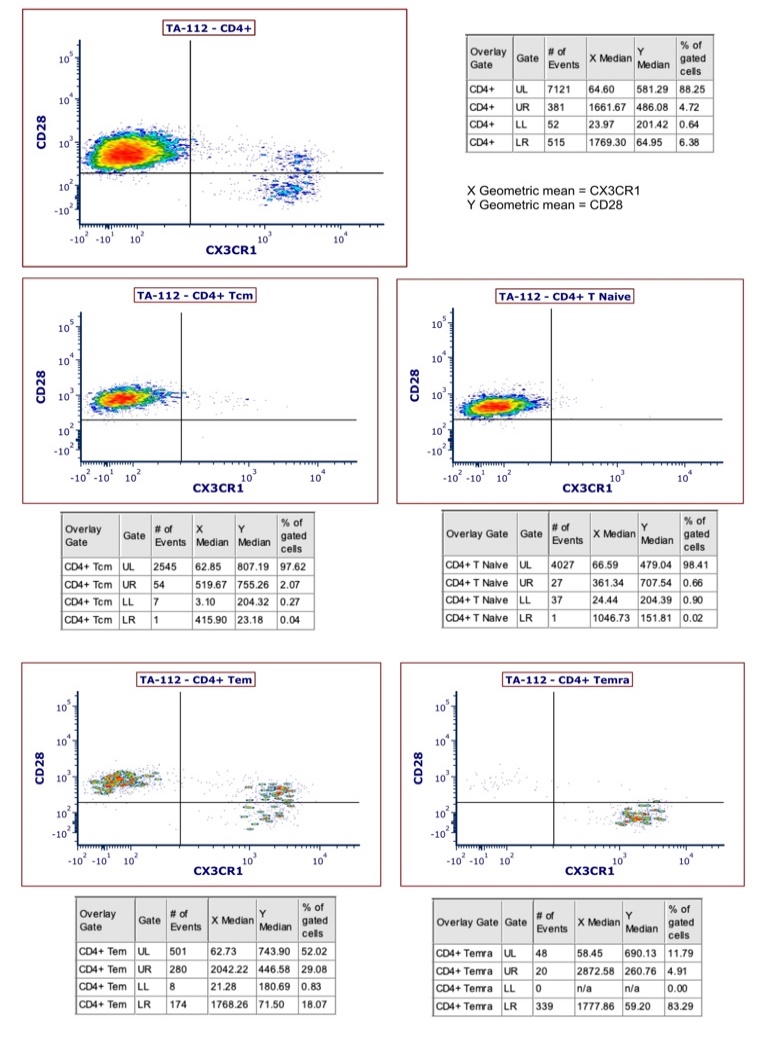

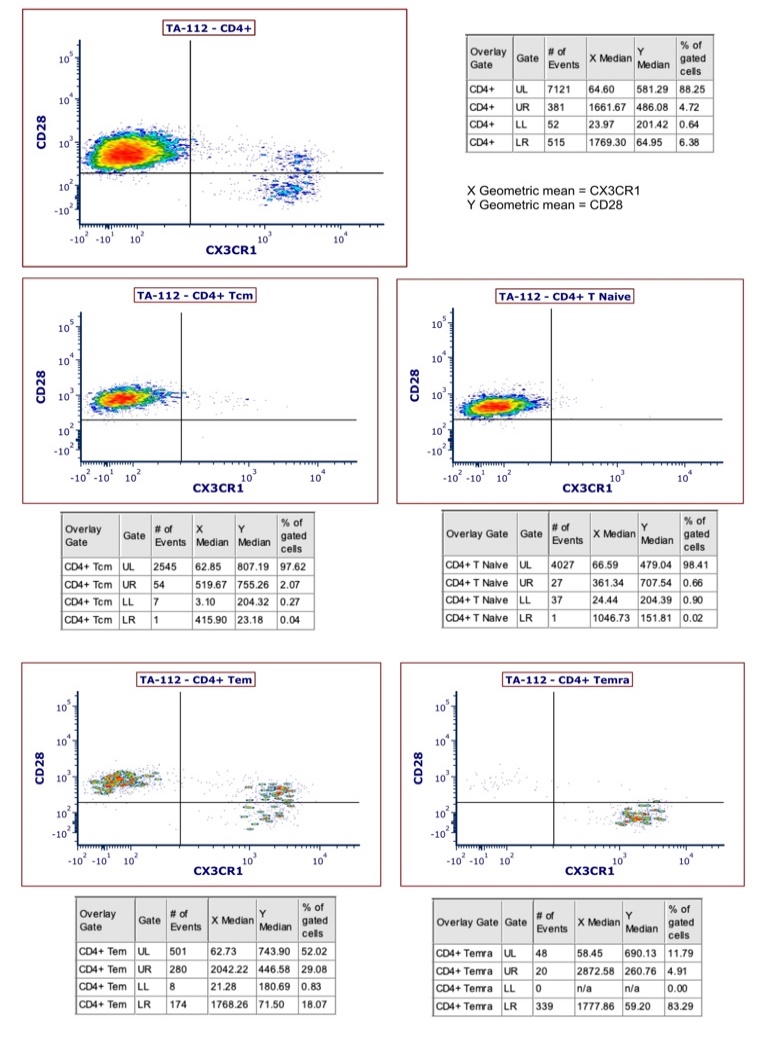

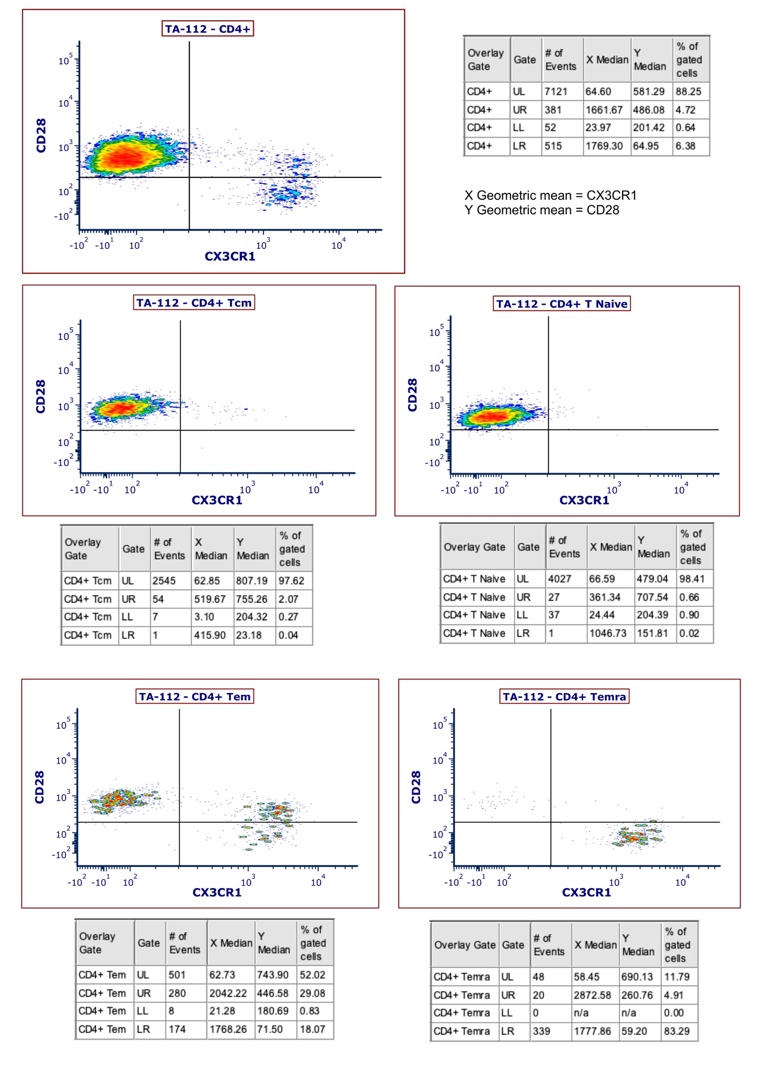

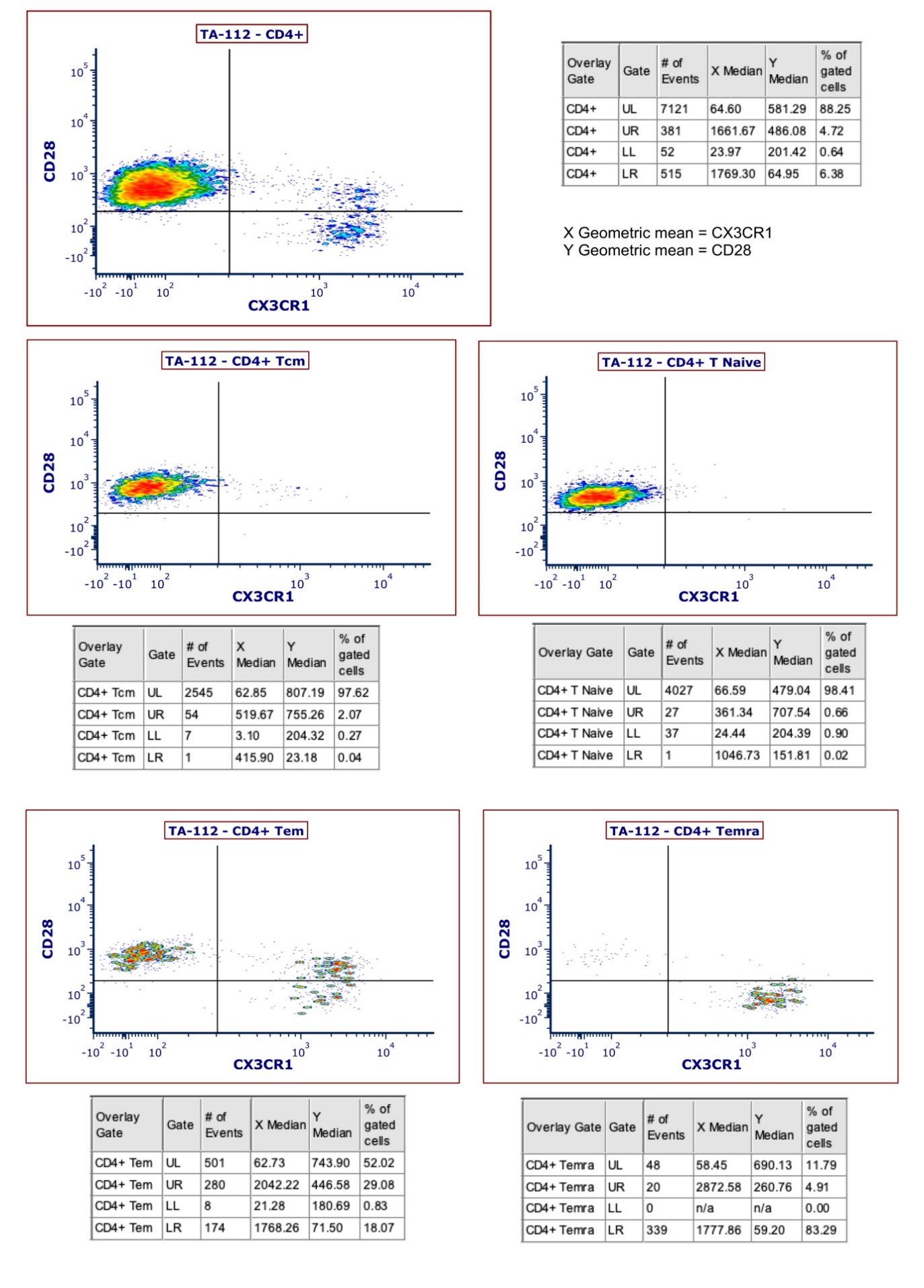

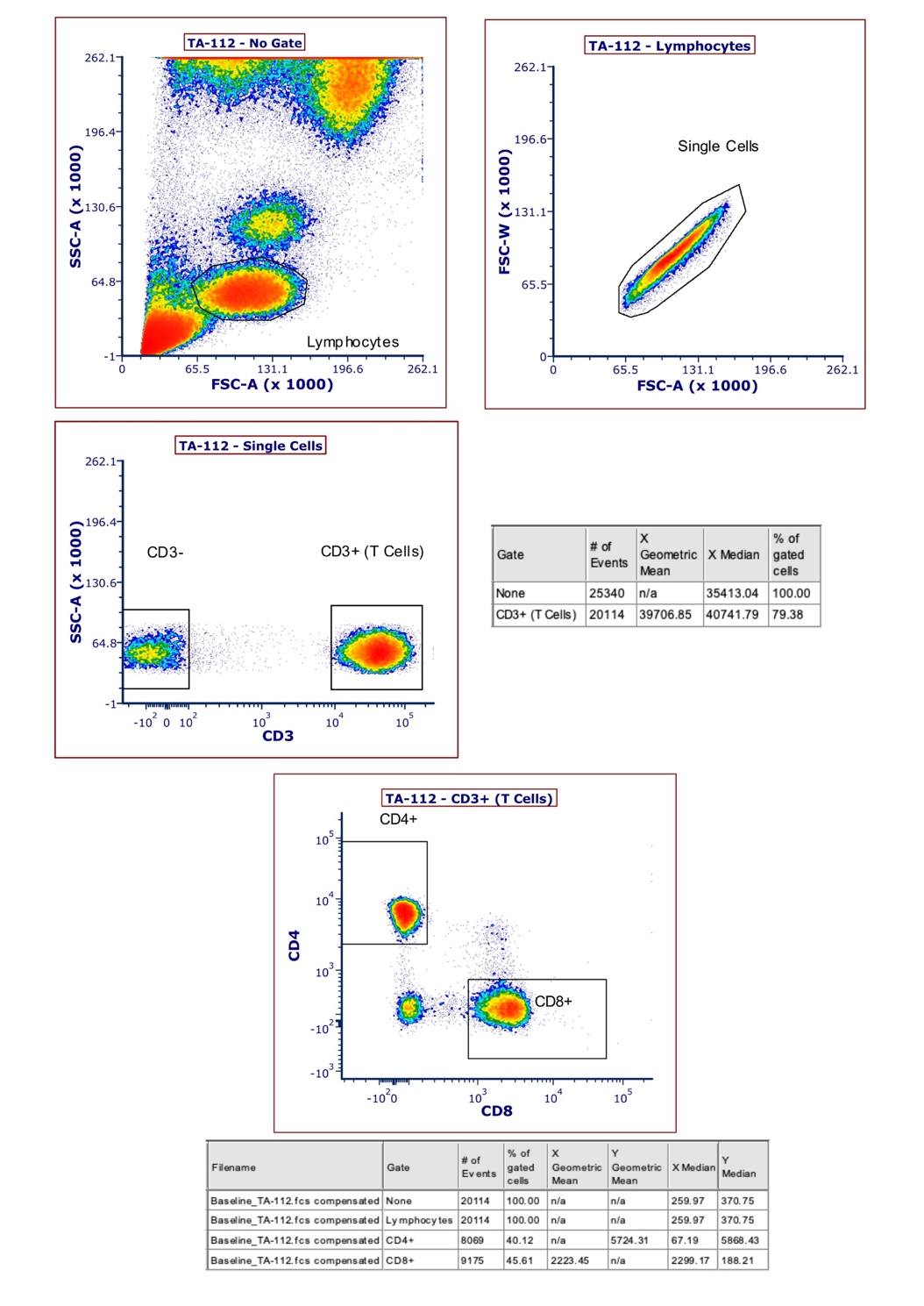

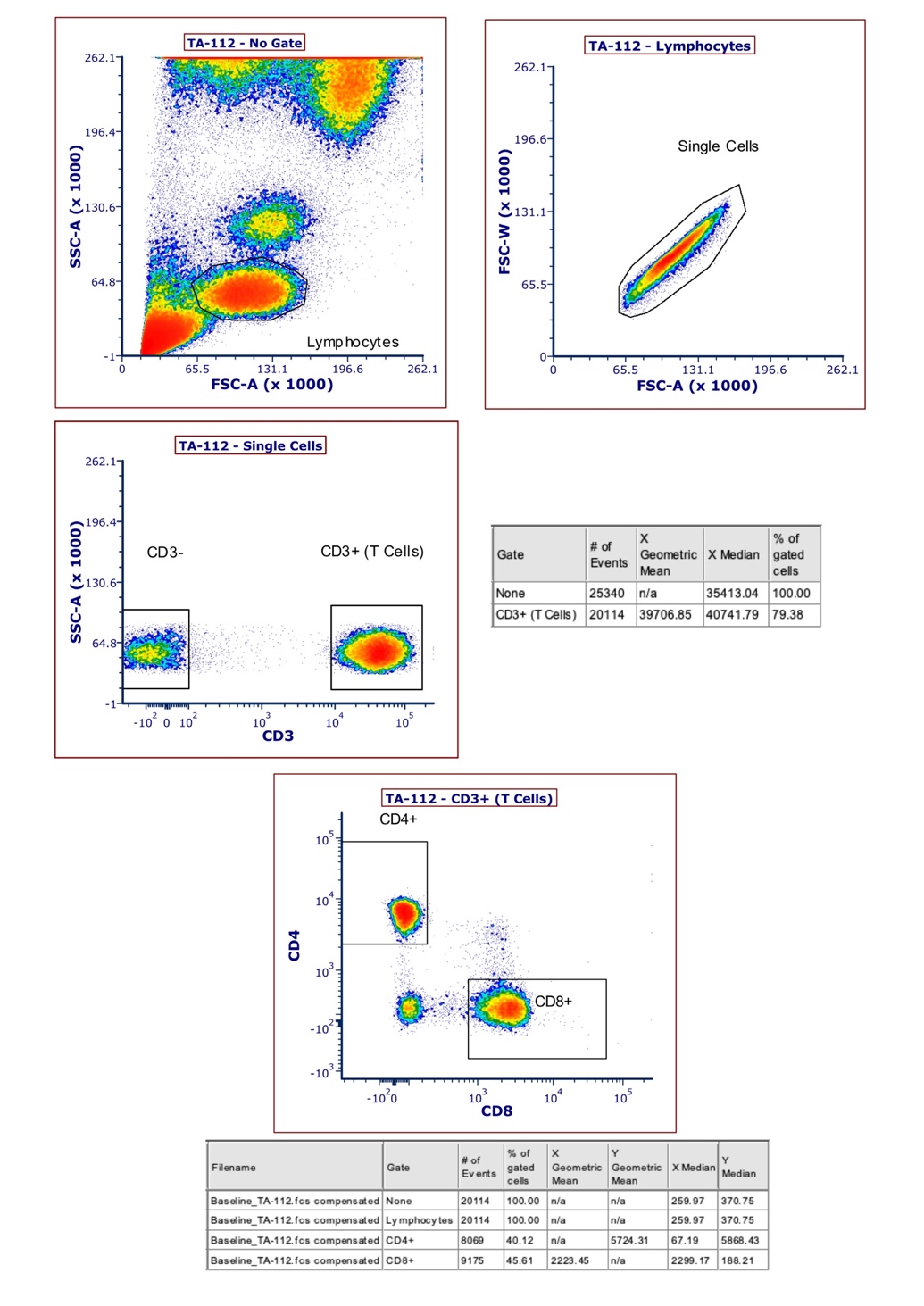

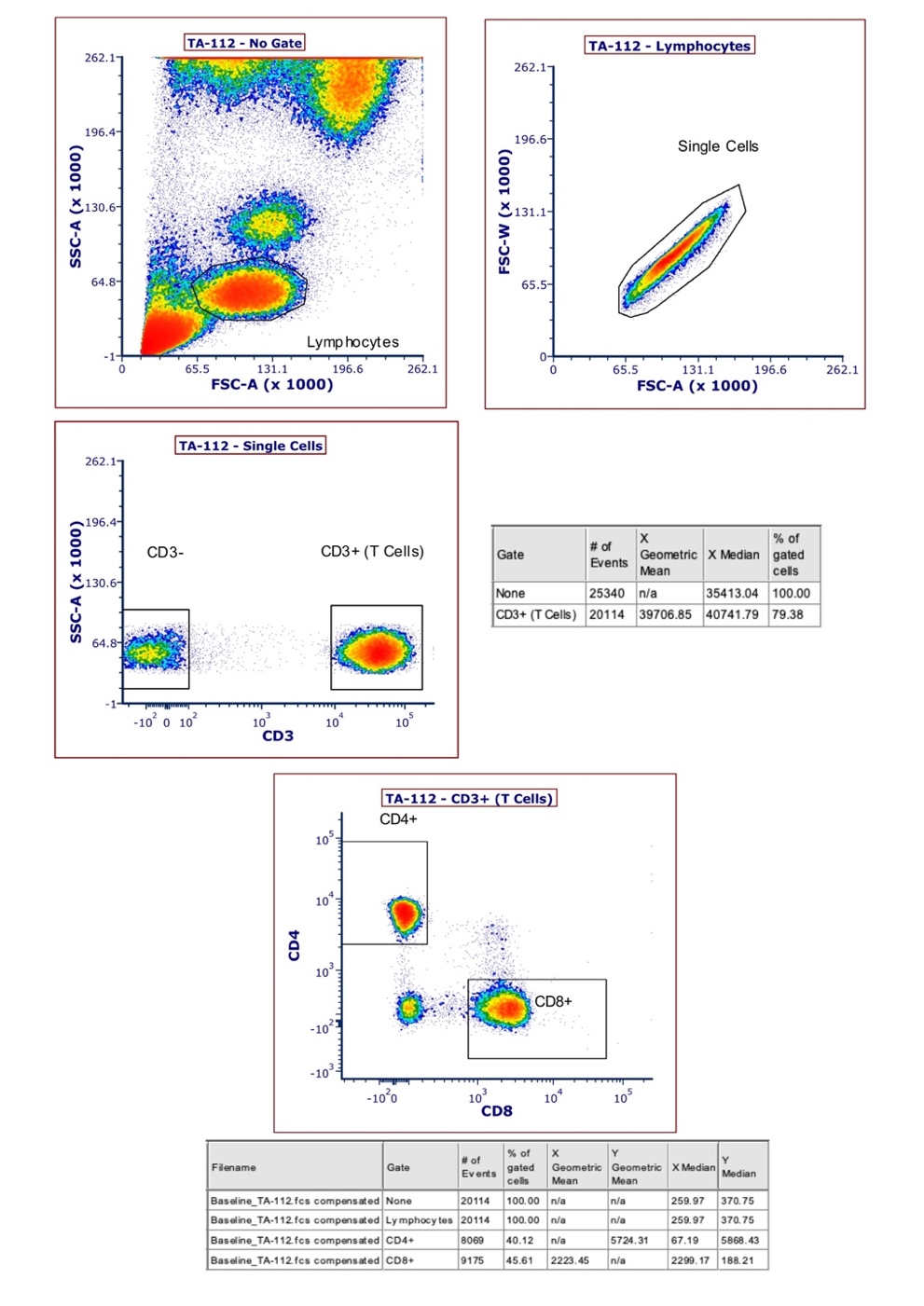

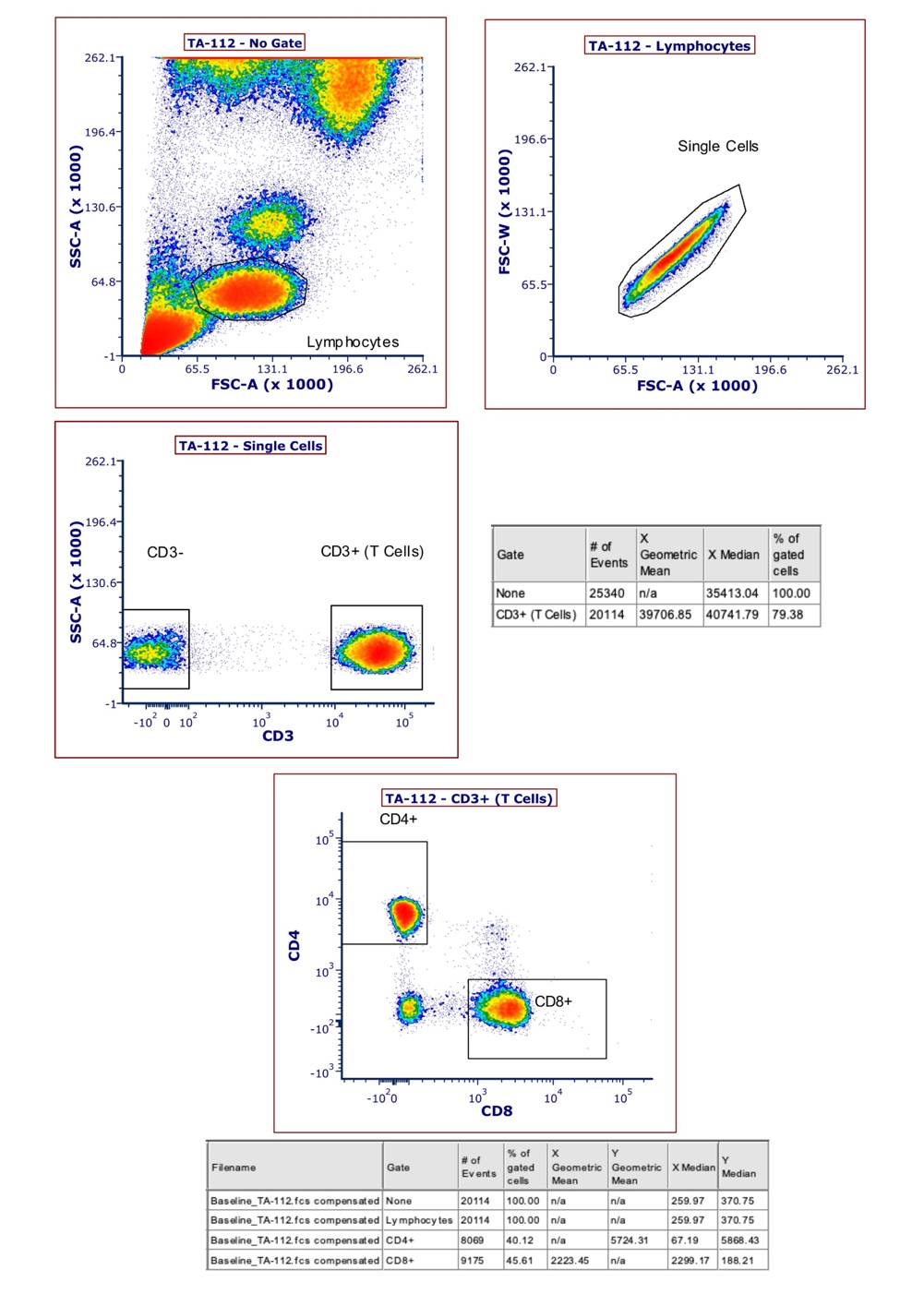

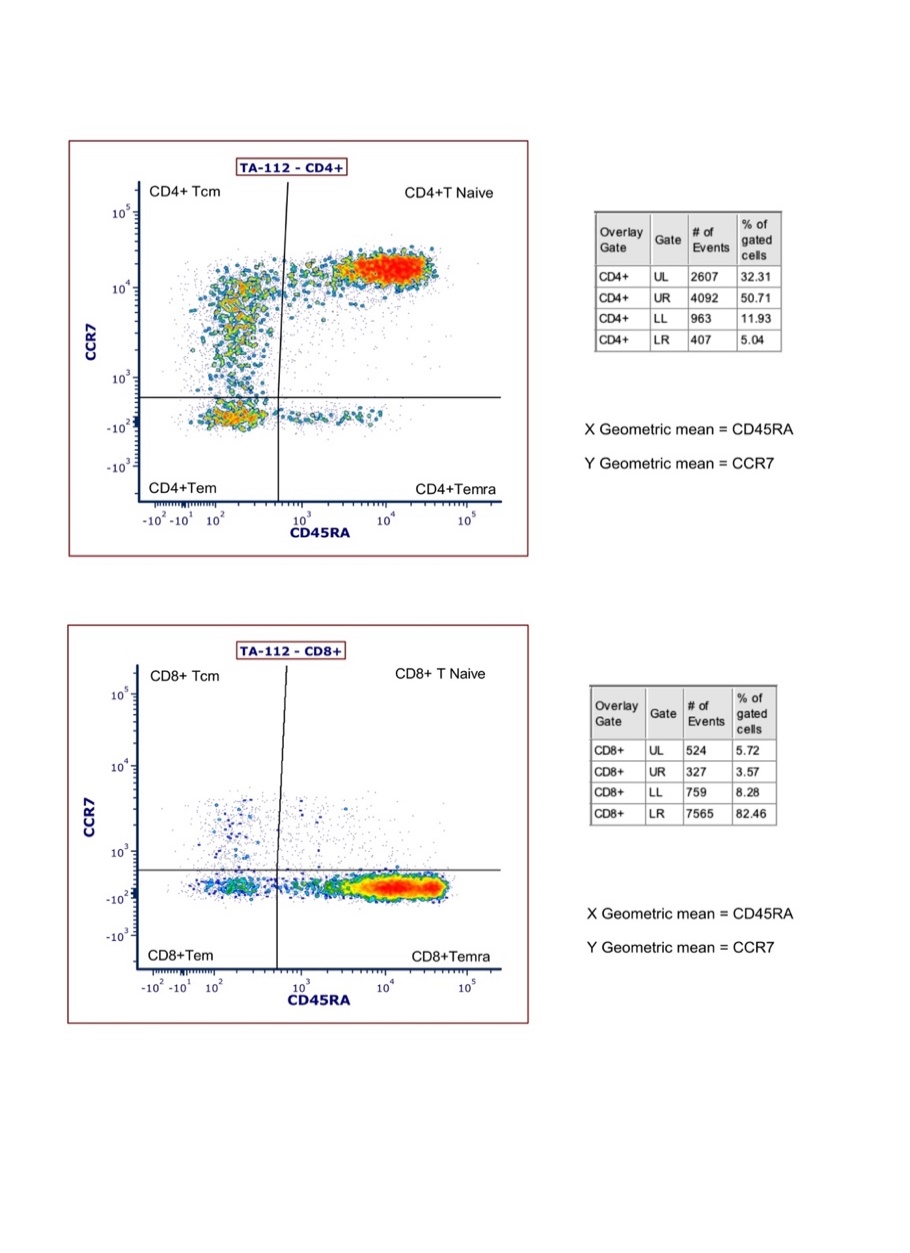

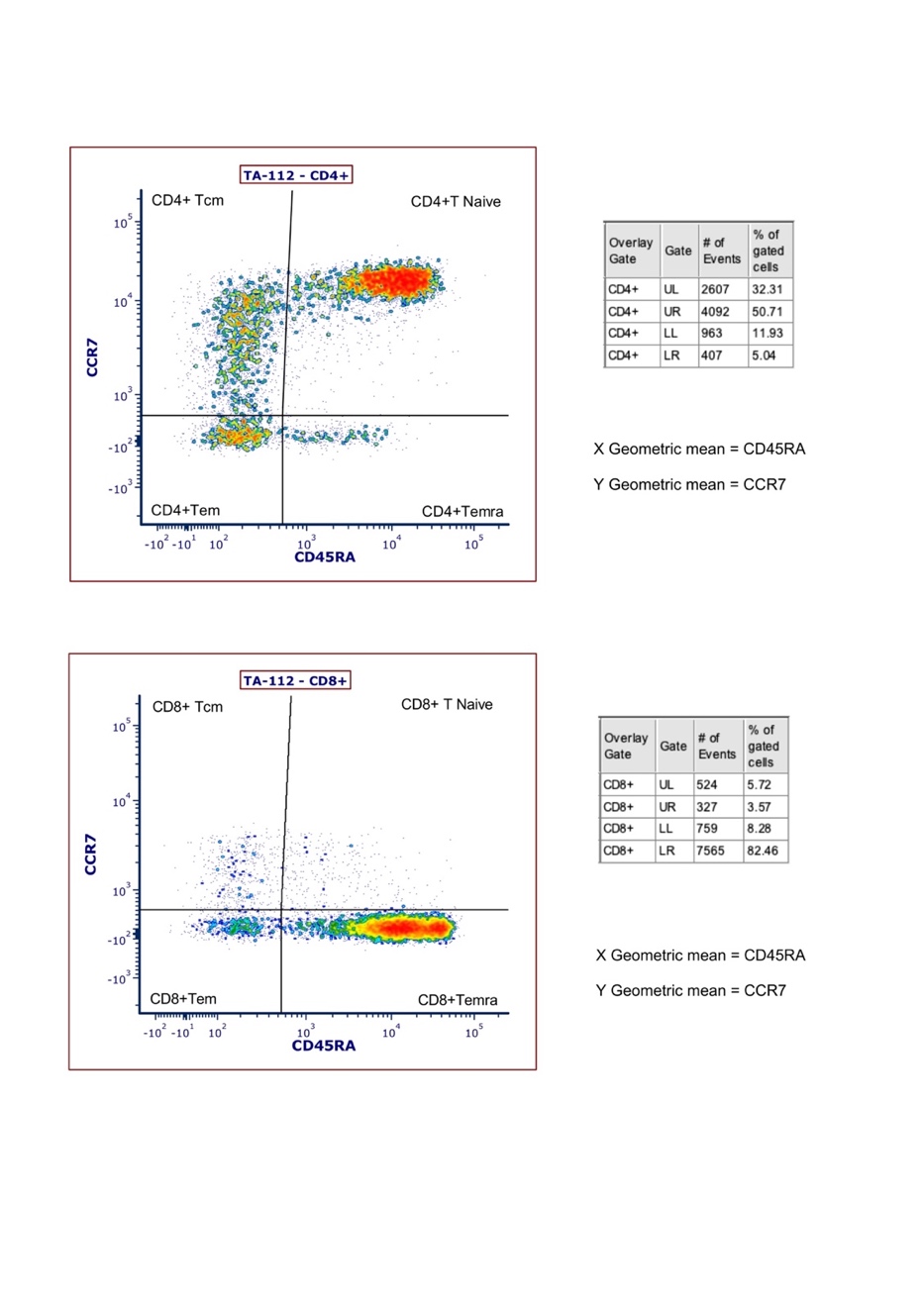

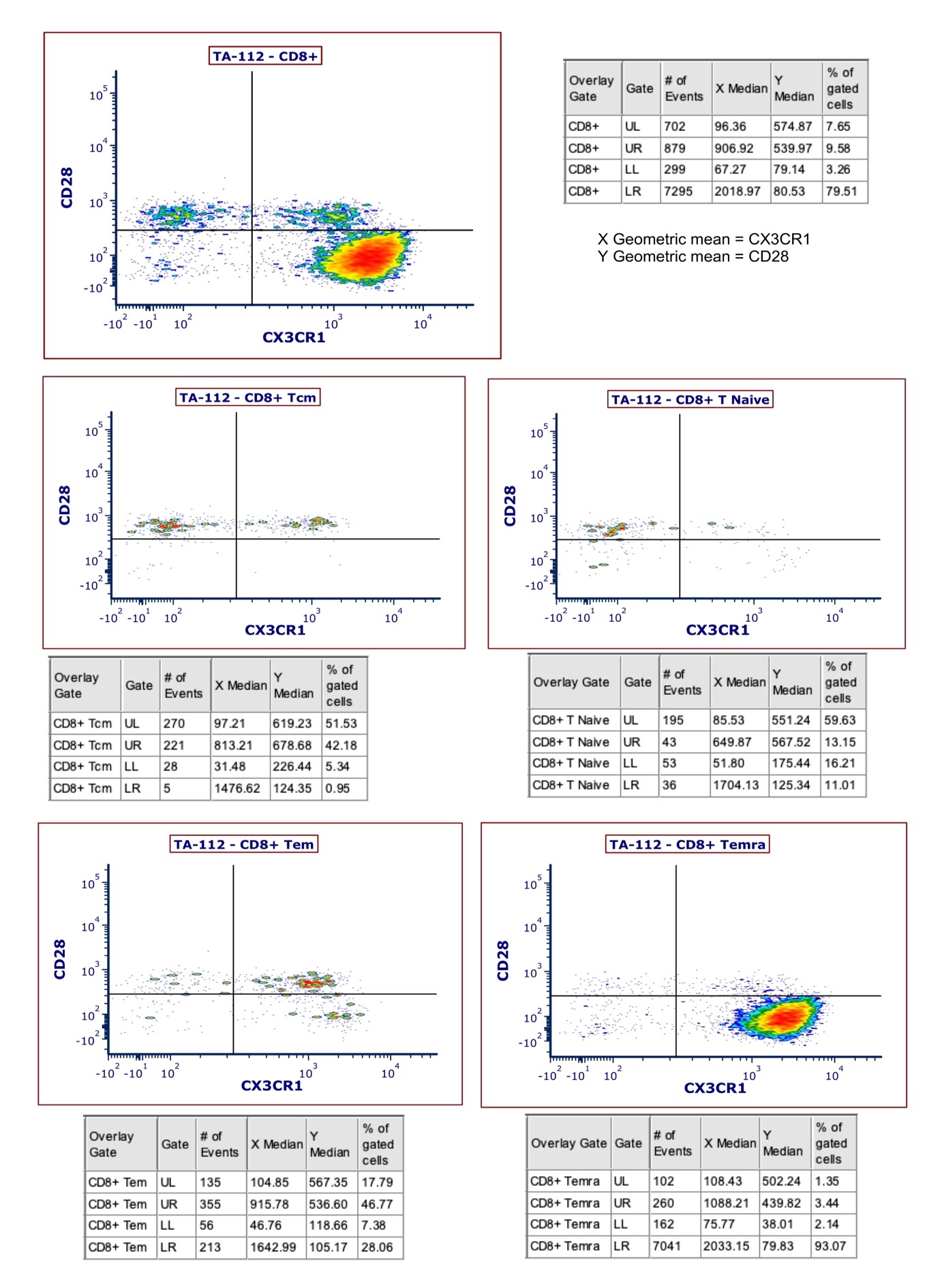

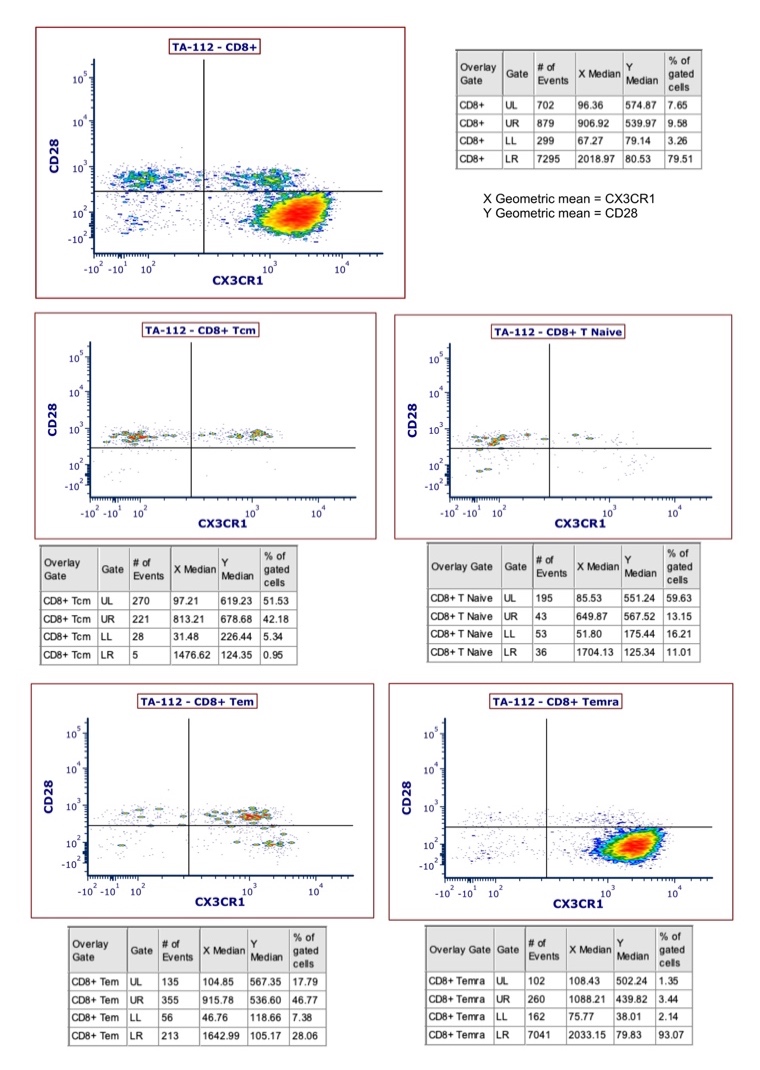

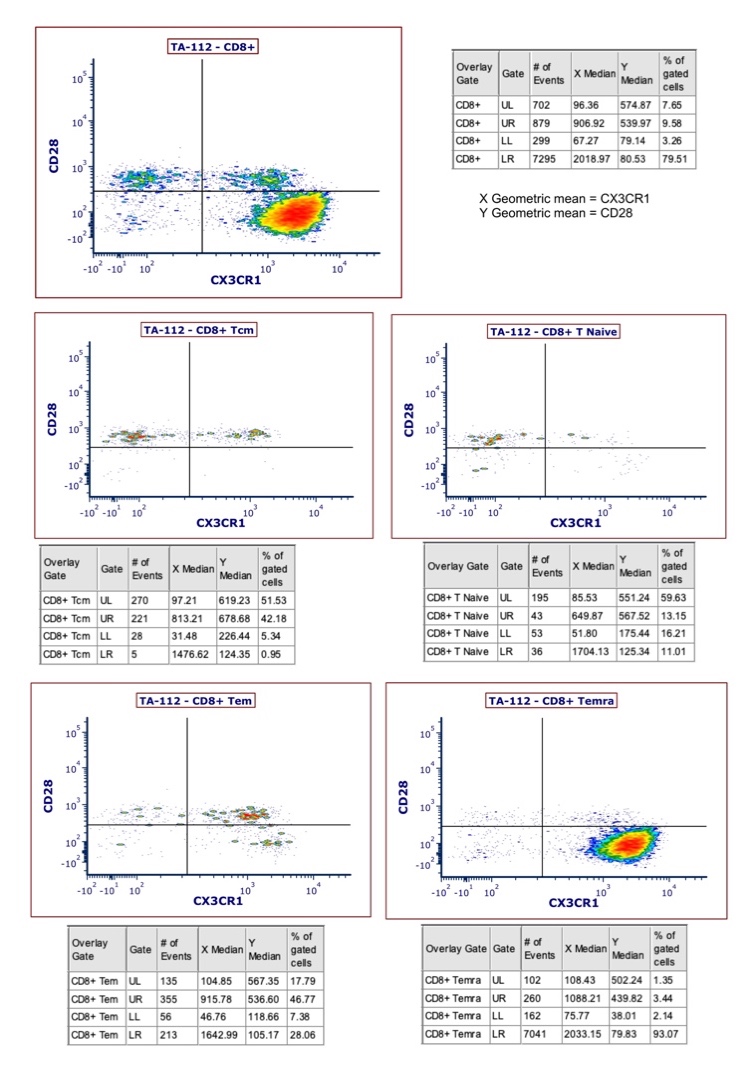

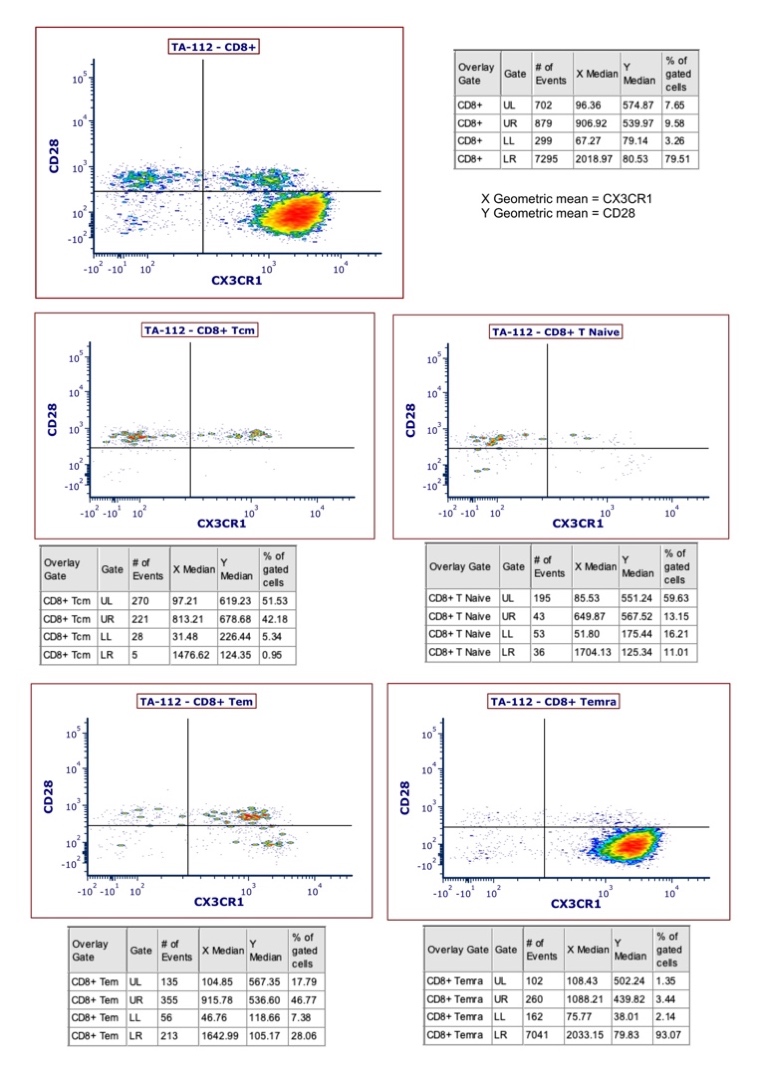

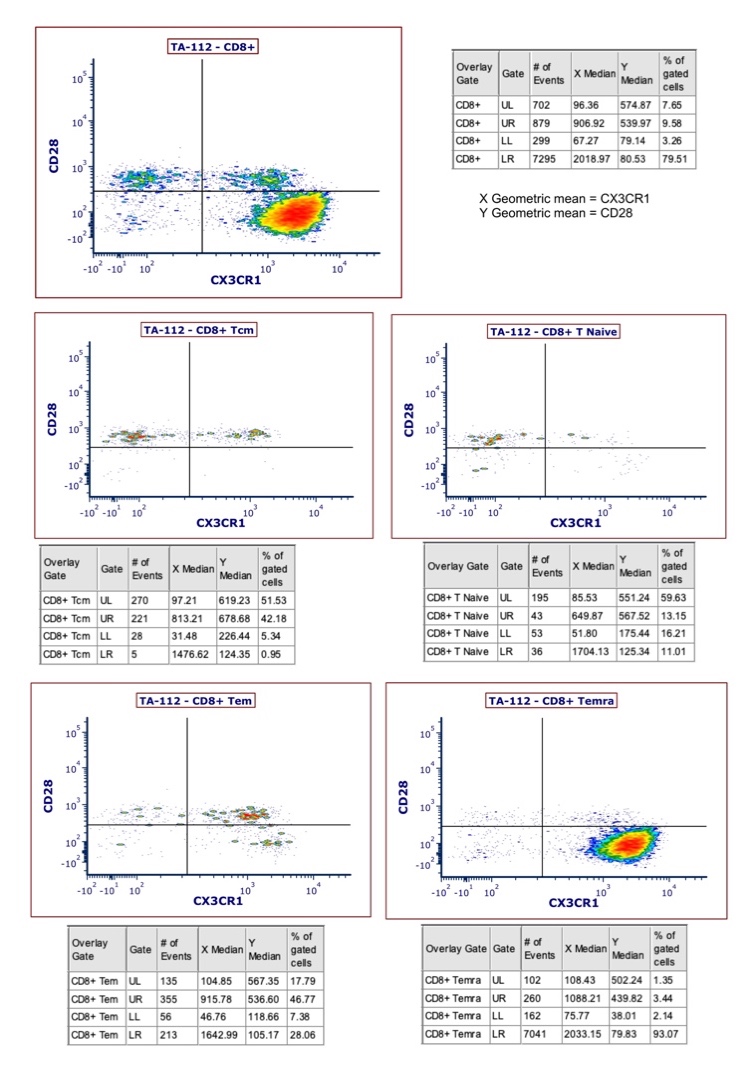


**Table S10 - Antibodies used for the 7-colour flow cytometric assay.** Catalogue number and manufacturer are given for each.

| Reagents for 7-colour assay | Clones | Volume (µL) | Catalogue Number (Manufacturer) |
| --- | --- | --- | --- |
| CD3-PE | UCHT1 | 20 | 555333 (BD Biosciences) |
| CD4-V500 | RPA-T4 | 5 | 560768 (BD Biosciences) |
| CD8-FITC | RPA-T8 | 20 | 555366 (BD Biosciences) |
| CD45RA- PE-Cy^TM^7 | L48 | 5 | 337186 (BD Biosciences) |
| CCR7-BV421 | G043H7 | 5 | 353208 (BioLegend) |
| CD28-AF700 | CD28.2 | 5 | 302920 (BioLegend) |
| CX3CR1-APC | 2A9-1 | 5 | 341610 (BioLegend) |

**Telomerase Repeated Activation Protocol (TRAP)**

Telomerase activity was measured using the Telomerase Repeated Activation Protocol (TRAP) – quantitative polymerase chain reaction (qPCR) assay. It is a PCR-based method that involves 3 basic steps:

1. Extension of an oligonucleotide by telomerase-mediated addition of telomeric DNA repeats
2. Subsequent PCR amplification of the extension products
3. Detection of telomerase products

Within 24 hours of collection, whole blood was centrifuged to isolate peripheral blood mononuclear cells which were cryopreserved at -80 C. The cells were subsequently thawed, lysed, and analysed using a validated qPCR protocol

***Isolating PBMCs***

Within 24 hours of collection, whole blood was centrifuged at 800G for 15 minutes to isolate peripheral blood mononuclear cells (PBMCs). These were cryopreserved at -80^o^C until being thawed for cell lysis and analysis.

***Positive control***

The human T-cell leukaemia cell line 1301 (Sigma-Aldrich) was used as a positive control during qPCR, as these cells have unusually long telomeres.

***Cell lysis***

Cryopreserved PBMCs (or 1301 cells for the positive control) were thawed and added to 9mL of phosphate-buffered saline (PBS; pH 7.2; 20012027, Gibco). This suspension was mixed, and 10µL was pipetted onto each side of a haematocytometer for counting. The cells were counted on each side and an average taken. The volume of suspension which would contain 2x10^6^ cells was calculated, pipetted out and centrifuged at 300-400G for 5 minutes to form a pellet.

The supernatant was then removed, and 500µL of PBS was added. The contents were resuspended and added to an Eppendorf tube. These were centrifuged for 8 minutes at 300-400G, with the tubes turned halfway through, to ensure complete lysis. The supernatant was carefully removed without disturbing the pellet of PBMCs. 40mL of lysis buffer 1x concentrate was added to the pellet to achieve a cell concentration of 50,000/µL. Lysis buffer was produced with BD Pharm Lyse Lysing Buffer (10x Conc. 5075567, BD Biosciences) and Gibco distilled water (15230-147, Life technologies). This was mixed well, then stored on ice for 30 minutes. This was then centrifuged at 4^o^C for 20 minutes at 12,000G. These were aliquoted into 5µL PCR tubes and stored at -80^o^C until qPCR analysis.

***TRAP-qPCR***

Samples (and positive controls) were removed from the freezer, and briefly centrifuged again. One 5µL tube for each sample (including positive control) was heat inactivated by incubating at 85 degrees for 10 minutes.

45µL of nuclease-free water was added to all samples, including the positive control, to achieve a cell concentration of 5,000/µL. The positive control was then twice diluted again 5-fold with nuclease-free water, to give final concentrations of 5,000, 1,000 and 200 cells/µL for the positive control.

2µL of sample were added to each well of a 96-well qPCR plate. Four replicates of each active sample and positive control were used, along with two replicates each for heat inactivated samples and lysis-buffer only samples.

The qPCR master mix was made with the reagents shown in **Table S11**, with volume shown per sample. This was made in batches to reduce pipetting error. The telomerase primer stock solution contained 10µL of 100ng/µL stock of both TS (telomerase substrate) primer (5’-AATCCGTCGAGCAGAGTT) and ACX primer (5’-GCGCGGCTTACCCTTACCCTTACCCTAACC-3’) diluted in 80µL nuclease free H_2_O.

18µL of master mix was added to each well. The plate was gently vortexed using a Vortex Genie 2 (Scientific industries), then centrifuged at 300-400G. The pPCR was then run with conditions as shown in **Table S12**, using a QuantStudio 7 Flex qPCR machine.

***Validation***

This protocol was validated through analysis of 8 quantities of human T-cell leukaemia cell line 1301 and generation of a standard curve. Serial 1:5 dilutions were used to give cell numbers ranging from 1 million to 13. The standard curve showed strong correlation (r^2^ = 0.94) and a slope value of -3.3, suggesting 100% efficiency (**Figure S2**). We found that telomerase activity was lower in PBMCs from patients in TACTIC than in the positive control (**Figure S3**). We therefore exclusively used 10^6^ cell samples for this assay with PBMCs from patients.

**Table S11 – Reagents used for TRAP-qPCR assay.**

| Reagent | Volume per sample |
| --- | --- |
| Nuclease-free H_2_0 | 4.8µL |
| Telomerase primer stock solution | 1.6µL |
| FastStart SYBR Green Master 2x concentration (Sigma-Aldrich #46734001) | 10µL |
| EGTA (10mM) | 2µL |

**Table S12. Conditions for TRAP-qPCR assay**.

| Step | Temperature (^o^C) | Time | No. cycles |
| --- | --- | --- | --- |
| Telomerase activation | 37 | 15 minutes | 1 |
| Telomerase inactivation, polymerase activation | 95 | 10 minutes | 1 |
| Denaturing | 95 | 20 seconds | 36 |
| Annealing | 52 | 20 seconds |  |
| Extension | 72 | 45 seconds |  |
| Data acquisition | Plate read | |  |
| Melt curve analysis | 95 | 15 seconds | 1 |
|  | 60 | 1 minute |  |
|  | 95 | 15 seconds |  |
|  | Plate read | |  |

**Figure S2. Standard curve for TRAP-qPCR assay using human T-cell leukaemia cell line 1301.** Shown are 8 serial 1:5 dilutions, starting at 1x10^6^ cells. There is a strong correlation (r^2^ = 0.94; p<0.001), and the slope value of -3.3 suggests 100% efficiency of the PCR.


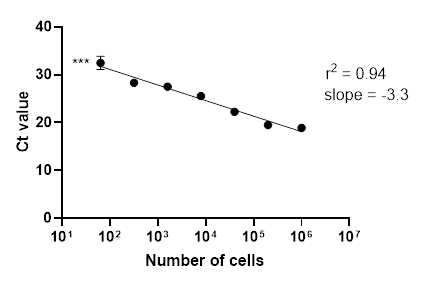


**Figure S3.** **Reproducibility of TRAP-qPCR results in TACTIC patients and positive control.** Serial 1:5 dilutions (starting with 50,000 cells) for the positive control (cell line 1301) and PBMCs from TACTIC patients 174 and 296 (two samples for each, A and B). In PBMCs from TACTIC patients, there was lower telomerase activity and a steeper slope of the calibration curve. All standard curves showed excellent reproducibility with r^2^ ≥0.99. Slope value for positive control was -3.37, and slope values for TACTIC patient samples were -5.74 (174A), -5.38 (174B), -5.43 (296A) and -5.31 (296B). Error bars are too small to be shown

.

**TBARS measurement**

Lipid peroxidation oxidative stress was measured using thiobarbituric acid-reactive substances (TBARS ) Assay Kit, Cat# KGE013, R&D, Minneapolis, USA. For precipitating interfering proteins and other substances, all the plasma patient samples were treated with trichloroacetic acid (TCA), with 1:1 ratio of 200ul plasma added to 200 ul of TCA, then mixed well. After 15 min incubation at room temperature, the samples were centrifuged at 12000 x g for 4 min, then 150ul of supernatants were incubated with 75ul of thiobarbituric acid (TBA) on a thermoshaker at 45 °C for 3 hours. The resulting color was measured spectophotometrically by determining the optical density of each well containing the patient plasma sample using Varioskan™ LUX (Thermo Scientific) multimode microplate reader at 532 nm wavelength.

**Measurement of endothelial function**

The EndoPAT device (Itamar Medical Ltd, Caesarea, Israel) was used to evaluate endothelial function by automatically calculating the reactive hyperaemia index (RHI) in an operator-independent manner. RHI is the post to pre occlusion PAT (peripheral arterial tone) signal ratio in the occluded arm relative to the same ratio in the control arm corrected for baseline vascular tone.

RHI has been shown to reflect NO-bioavailability (1). RHI lower than 1.35 was 80% sensitive and 85% specific in identifying patients with coronary endothelial dysfunction (2) and RHI values are predictive of cardiovascular outcomes (3). Moreover, low RHI can be reversed with treatment (4).

Patients were seated in a quiet and comfortable room for at least 15 minutes before the start of the test. Blood pressure was measured from the patient’s control arm. The PAT probes were inserted in each index finger and a blood pressure cuff placed on the upper arm of designated test arm. Both PAT probes were connected by pneumatic tubes to an inflating device controlled by a computer running the EndoPAT software.

The PAT signals from both test and control arms were visualised and inspected for strength and stability. Baseline signals were recorded for 5 minutes. The blood pressure cuff was rapidly inflated and maintained at supra-systolic level (at least 60 mmHg above systolic blood pressure) for 5 minutes. Total cessation of blood flow to the test arm was verified by the total absence of the PAT signal. The blood pressure cuff was rapidly deflated and post occlusion PAT signals recorded for 5 minutes.

Following the recording period, the RHI was obtained using the automatic analysis function on the software (**Figure** **S4**). Occlusion borders are reviewed and adjusted manually if appropriate.

**Figure S4: Automatic analysis of RHI (reactive hyperaemia index) using pre and post occlusion PAT signals in control and test arms.**

**Echocardiography**

Patients underwent 2-dimensional (2D) transthoracic echocardiography at baseline and 12 months, using the Phillips Affiniti 70 and GE Vivid E95 ultrasound system. Measurements were performed in either a minimum of three or five cardiac cycles depending on the underlying cardiac rhythm (sinus rhythm or atrial fibrillation, respectively).

All echocardiographic images were digitally stored in DICOM format and analysed offline using TomTec™ (TOMTEC Imaging Systems, Unterschleißheim, Germany) software at the James Cook University Hospital Echocardiography Core Laboratory, by echocardiography accredited cardiology registrars (SV and BB).

Using the parasternal long axis view, 2D linear measurements of the left ventricular cavity dimensions were performed. Non-foreshortened apical 4- and 2- chamber views with a good endocardial definition were used for left ventricular volumetric assessment. Left ventricular ejection fraction was then quantified using the modified Simpson’s biplane measurement. Left ventricular end-systolic global longitudinal strain (GLS) was measured using TomTec™ semi-automated software (AutoSTRAIN^©^) after selection of non-foreshortened apical 4-, 2- and 3-chamber views with reasonable image quality and complete R-R cycles. Pulse waved spectral Doppler in the apical 5-chamber view was then used to allow the measurement of aortic valve closure time (AVC) for end-systolic GLS quantification. If automated endocardial tracings were deemed unsuitable adjustments to these tracings were made to allow accurate GLS measurement. If tracking of the endocardium was unsatisfactory, GLS was not measured.
